# Human Serum Albumin Loaded with Fatty Acids Reveals Complex Protein–Ligand Thermodynamics and Boleadora-Type Solution Dynamics Leading to Gelation

**DOI:** 10.1021/acs.jpcb.4c08717

**Published:** 2025-03-26

**Authors:** Jörg Reichenwallner, Sebastian Michler, Christian Schwieger, Dariush Hinderberger

**Affiliations:** † Institute of Chemistry, Physical Chemistry − Complex Self-Organizing Systems, Martin Luther University Halle-Wittenberg, Von-Danckelmann-Platz 4, 06120 Halle (Saale), Germany; ‡ Department of Biochemistry, University of Toronto, Toronto, Ontario M5S 1A8, Canada

## Abstract

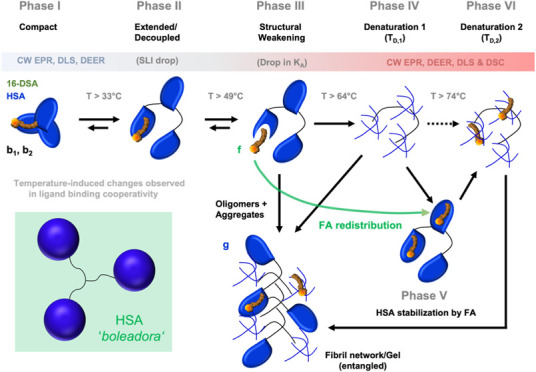

Using an electron paramagnetic resonance (EPR) spectroscopic strategy that has been developed for core–shell polymers, the complexity of the binding of fatty acids to human serum albumin (HSA) is characterized in detail. We unravel the internal dynamics of HSA solutions with fatty acids by applying continuous wave EPR (CW EPR) from which we derive a consistent thermodynamic interpretation about fatty acid interactions with HSA in the investigated temperature range of 5–97 °C. Additionally, data from CW EPR are corroborated by dynamic light scattering (DLS), differential scanning calorimetry (DSC) and nanoscale distance measurements using double electron–electron resonance (DEER) spectroscopy. We discuss our data in light of decades of biophysical studies on albumin and aim at drawing a complete functional and dynamic picture of HSA “at work”. This picture suggests that HSA is built from modular, rotationally decoupled domains that resemble an entangled three-piece *boleadora* in solution.

## Introduction

**Figure 1:**
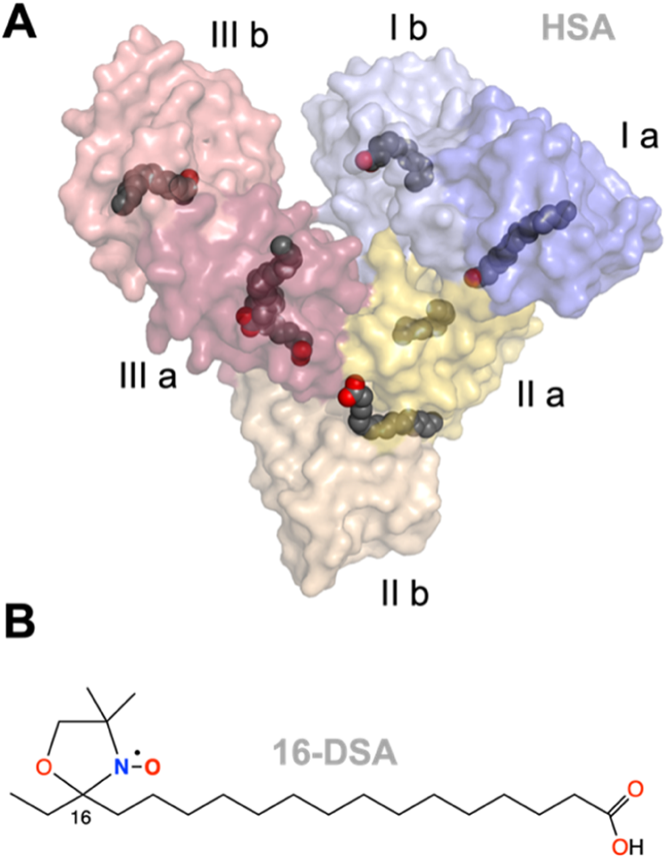
Structures of HSA and 16-DSA. (A) Crystal structure (PDB 1e7i) of HSA cocrystallized with seven stearic acid molecules.^45^ The oxygen atoms of the FA carboxylic acid head groups are displayed in red. (B) Chemical structure of the paramagnetic EPR-active ligand 16-DSA.

Many types of serum albumins, the major transport proteins in the blood of mammals, have been extensively studied in protein biophysical chemistry since the 1950s.^1−3^ Due to their high abundance (near mM concentration in blood) and availability, albumins of different species mainly bovine serum albumin (BSA) and to a much lesser degree human serum albumin (HSA) have been used as model proteins for ligand binding studies.^4,5^Although BSA and HSA share high sequence identity and similar X-ray crystal structures, they cannot be considered identical. Recent studies, including our own, have highlighted the unique individual properties of these proteins.^6−8^ Continuous wave electron paramagnetic resonance (CW EPR) spectroscopy has been used from early on to study fatty acid (FA)-albumin interactions. However, due to their enormous complexity, CW EPR spectra of spin-labeled fatty acids (SLFA) bound to albumin were predominantly treated phenomenologically in the 1970s and 1980s.^9−16^ Most of these approaches studied uptake capabilities and ligand interactions of albumin and paramagnetic FAs. Beyond these rather phenomenological studies, rigorous, simulation-based dynamic analysis of albumin associated with a variety of SLFAs were reported by Shenkar et al.^17^ and in an earlier attempt by Gaffney and McConnell.^18^ In recent years, spectral simulations of spin-probed or spin-labeled albumin samples have resurged, mainly due to broad accessibility of powerful spectral simulation tools for CW EPR.^8,19−36^ The main information content from CW EPR spectra on FA-probed HSA and their rigorous simulations is, first, the^14^N-hyperfine coupling (isotropic *a*_iso_ or hyperfine coupling tensor component *A*_*zz*_) to the electron spin density that gives insights into the environmental polarity with a lower *a*_iso_ value indicating nonpolar and a higher *a*_iso_ value a more polar environment. A second, even more important aspect that can be investigated is rotational diffusion, which gives us information on the motional freedom of a nitroxide (ligand).^3^ The latter is usually described by the isotropic rotational correlation time τ_C_. Standard CW EPR experiments on nitroxides are sensitive to changes in *a*_iso_ values from 40 to 48 MHz and rotational correlation times in the range from about 10 to 50.000 ps (50 ns). A scheme of FA alignment in HSA is shown in Figure 1A.

Here, we aim at crafting a complete model of the functional (solution) structure and dynamics of albumin as derived from its ligand binding. We will discuss the work presented here against the large body of remarkably inconsistent EPR-spectroscopic and other biophysical data to construct a model that robustly fits all major findings so far. In particular, in the pertinent literature one finds a range of overall 2–5 spectral components of SLFAs bound to albumin.^17,19,27^ This may be mainly due to problems with separability of the individual spectral components.^37^ Building on an analysis developed for characterization of FA uptake in core–shell polymeric systems,^38,39^ we here transfer this mathematical treatment to derive a physical picture of how FA assembly in HSA affects the protein’s structure and dynamics.

It has already been shown that albumin also exhibits two types of immobilized spectral components from the stearic acid derivative 16-DSA (16-DOXYL stearic acid, see Figure 1B) that are commonly assigned to weak and strong binding sites.^20,27,31,32^ As these components change their spectral fractions with temperature and FA loading, it was proposed that the occupation of weak and strong binding sites changes due to an intra-albumin migration mechanism with an apparent activation energy of 26.8 kJ/mol for HSA and 35.2 kJ/mol for BSA.^20^ EPR spectroscopic thermal denaturation studies based on spectral simulation of both, SLFA-probed HSA and BSA, have already been conducted in the range from 20 to 50 °C,^20,31^ however, well below their individual denaturation temperature *T*_D_. This is largely due to the impressive thermostability of HSA which is based on its highly conserved 17 disulfide bridges.^4,40^

In this study, we mainly report EPR-spectroscopic data of temperature-dependent HSA-FA binding and simultaneous HSA denaturation, allowing us to derive a picture of the local dynamics and global structure of the protein. To this end, CW EPR and double electron–electron resonance (DEER) data on HSA^3,6,41−44^ are complemented with other techniques such as differential scanning calorimetry (DSC) and dynamic light scattering (DLS) to reveal a solution-based dynamic model of albumin interacting with SLFA ligands in the temperature range of liquid water. Several well-known phenomena are recapitulated, discussed and integrated to corroborate a model reflecting that HSA in solution consists of modular, rotationally decoupled domains resembling a self-capturing boleadora-type domain alignment.

## Materials and Methods

### Materials

Lyophilized powder of HSA (>95%, Calbiochem), 16-DSA (Sigma-Aldrich) and 87 wt % glycerol (ACROS Organics) were used without further purification. The 0.137 M Dulbecco Phosphate-Buffered Saline (DPBS) buffer^46^ with pH 7.4 was prepared according to the procedure described in the Supporting Information S1.

### Sample Preparation

All experiments were conducted on HSA solutions loaded with 16-DSA equivalents of moderately varying ratios. Regardless of the applied method the protein concentration was kept constant at about *c*_HSA_ = 0.18 mM. As the stability of HSA is also dependent on its concentration, only the concentration of 16-DSA was varied by default throughout the whole study. All sample volumes were adjusted for device-specific requirements ranging from about 0.01–1.00 mL. HSA was dissolved in 0.137 M DPBS buffer pH 7.4 to a final stock concentration of 1 mM. Upon addition of an 8 mM stock solution 16-DSA dissolved in 0.1 M KOH the solution gained a slight alkaline pH change being compensated by titrating with appropriate alkaline and acidic 0.12 M DPBS-buffers (range pH 1–13) containing varying amounts of HCl and NaOH, so that final physiological values of pH 7.42 ± 0.06 could be comfortably adjusted for all samples. The exact amounts of added 16-DSA was determined by double integration of corresponding CW EPR spectra throughout.

**Figure 2:**
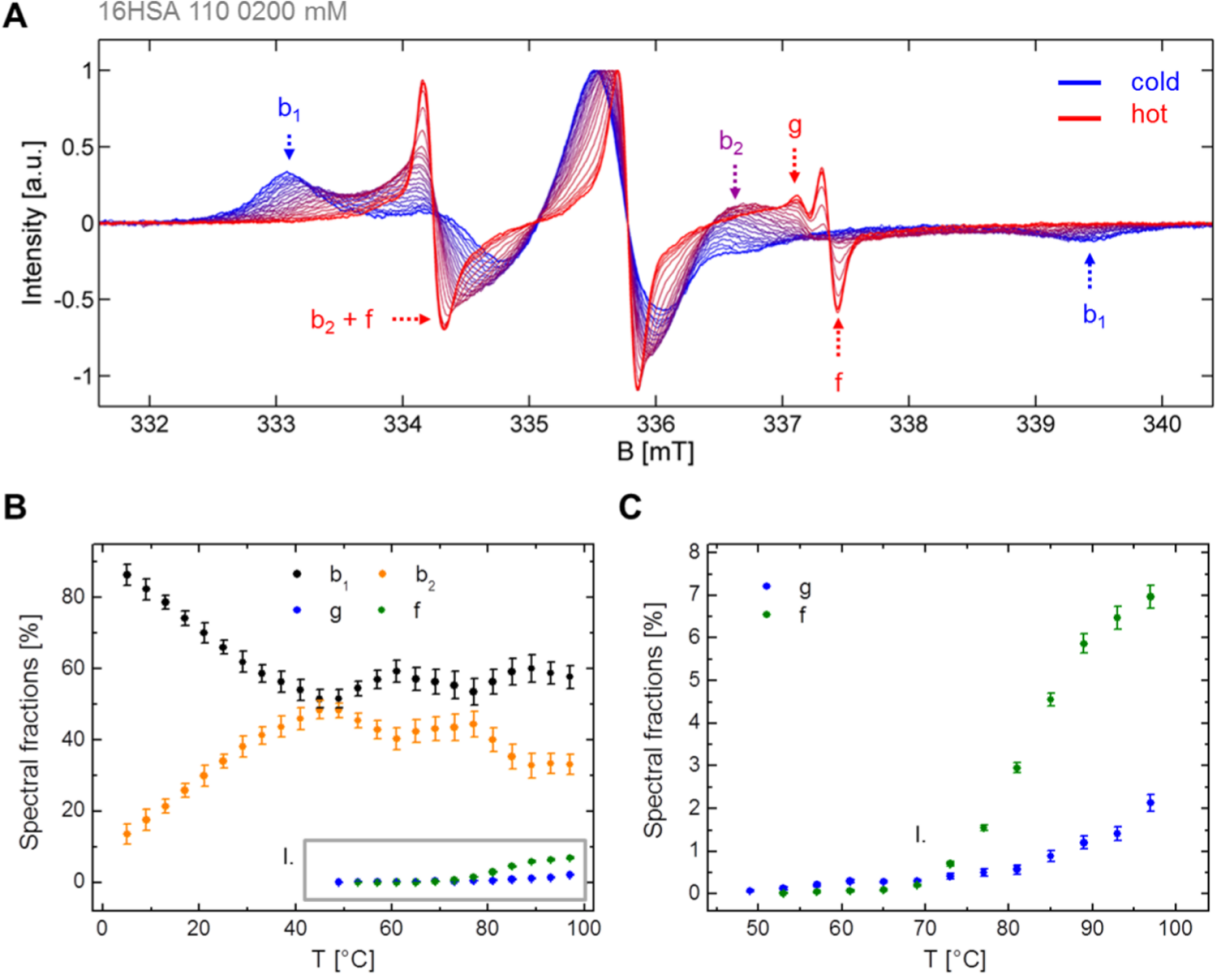
Temperature-dependent CW EPR spectra of 16-DSA interacting with HSA. Spectra were measured in the temperature range from 5 to 97 °C in steps of Δ*Τ* = 4 °C. The exact 16-DSA loading ratio of HSA is 1.13:1.00 (*c*_HSA_ = 0.18 mM). (A) CW EPR spectra of 16-DSA interacting with HSA. The most prominent spectral features of dynamic fractions *f*, *b*_1_, *b*_2_ and *g* are highlighted. The lowest (dark blue) and the highest (dark red) temperature curves are shown in bold to create an envelope effect. (B) Spectral fractions from simulations of 16-DSA interacting with HSA. Immobilized fractions are shown in black (*b*_1_) and orange (*b*_2_), whereas hydrogel-like fractions (*g*) are shown in blue and free fractions (*f*) are shown in green. (C) A magnification of inset I in (B) is shown. All error margins have been determined from individual spectral simulations.

Temperature steps for HSA denaturation with CW EPR (Figure 2) were set to Δ*T* = 4 K and the molar ratio of HSA to 16-DSA was 1.00 to 1.13 with a 16-DSA concentration of [*L*]_*t*_ = 204 ± 6 μM. About 15 μL of the final solutions were filled into appropriate EPR-silent capillaries (BLAUBRAND IntraMARK) for the experiment.

In order to determine temperature dependent association constants *K*_A*,j*_ and number of equivalent binding sites *N*_E,*j,p*_ from CW EPR-based Scatchard plots, the molar ratios of HSA to 16-DSA were varied in the range from 0 to 8 equiv at pH 7.4 to prevent ligand micelle formation.^13,47^ The critical micelle concentration (CMC) of 16-DSA was determined as (285 ± 29) μM via CW EPR spectroscopy (see Supporting Information S2). Micelles are thus expected when the free ligand concentration [L]_*f*_ would exceed about 0.3 mM. This unwanted phenomenon was successfully prevented in all recorded CW EPR spectra. Likewise, for DEER spectroscopy it is crucial that no micelle-based exchange interaction can occur between proximal nitroxide moieties to maximize dipolar evolution time and thus achieve maximum data quality.

Unlike in CW EPR experiments of this study, all DEER samples were additionally equipped with 20% v/v glycerol to prevent crystallization upon freezing. The same accounts for individually prepared samples that were used in the 16-DSA loading study with DEER for extraction of reference data for the DEER temperature denaturation. For temperature denaturation as monitored by DEER, a single sample was prepared as a 1 mL stock solution at pH 7.38 that was aliquoted into 0.05 mL fractions, so that each sample contains identical ingredients and conditions. All spin probed HSA samples for DEER spectroscopy were filled into 3 mm outer diameter quartz tubes (Heraeus Quarzschmelze) and the aliquots were additionally incubated for 5 min at individual temperatures in the range from 9 < *T* < 81 °C. The incubation temperatures for DEER experiments were chosen to coincide with temperatures in all other experiments and were also conducted in steps of Δ*T* = 4 K in a water bath that was set up with ultrapure water (Milli-Q) in Eppendorf reaction tubes being preheated for sufficient time in an Eppendorf Thermomixer C (∼5 min for each temperature). Directly after temperature incubation, the samples were shock frozen in liquid nitrogen-cooled 2-methylbutane (Sigma-Aldrich) for subsequent DEER measurements. The samples for mere 16-DSA loading studies with DEER were conveniently just shock frozen from room temperature without further treatment.

### EPR Spectroscopy

#### CW EPR Experiments

A Miniscope MS400 (Magnettech GmbH) benchtop spectrometer was employed for X-band CW EPR measurements operating at a microwave frequency of 9.4 GHz. All measurements were performed in the temperature range of 5 < *T* < 97 °C utilizing modulation amplitudes of 1 G during a field sweep of 15 mT with an incident microwave power in the range of 3.16 mW. For precise temperature adjustments (intrinsic error is about 0.3 K) a temperature controller (Magnettech Temperature Controller H03) was used. EPR spectra for 16-DSA-based temperature response curves of HSA were recorded in steps of Δ*T* = 4 K with a precautionary incubation time for each temperature step of about 2–5 min. The microwave frequency was recorded with a frequency counter (RACAL-DANA, model 2101).

#### DEER Experiments

The four-pulse DEER sequence:^48,49^ ± (π/2)_obs_–τ_1_–(π)_obs,1_–*t*_d_–(π)_pump_– τ_2_–(π)_obs,2_–τ_2_– RE was used to obtain dipolar time evolution data from paramagnetic 16-DSA spin probes interacting with HSA at X-band frequencies of 9.1–9.4 GHz with a BRUKER Elexsys E580 spectrometer equipped with a BRUKER Flexline splitring resonator ER4118X–MS3. The temperature was set to *T* = 50 K by cooling with a closed cycle Helium cryostat (ARS AF204, customized for pulse EPR, ARS, Macungie, PA) and the resonator was overcoupled to ca. *Q* ≈ 100. The pump pulse position after the first observer π-pulse deadtime *t*_d_ was typically incremented for *N*_*t*_ timesteps of Δ*t* = 8 ns in the range τ_1_ + τ_2_ – 2*t*_d_, whereas τ_1_ and τ_2_ were kept constant. Proton modulation was averaged out by summation of eight time traces of variable τ_1_ starting with τ_1,1_ = 200 ns, incrementing by Δτ_1_ = 8 ns and ending up at τ_1,8_ = 256 ns. Additionally, a 2-step phase cycle (±) was applied to the first π/2 pulse of the observer frequency for canceling out receiver offsets and unwanted echoes. The pump frequency ν_pump_ was set to the maximum of the field swept electron spin echo (FS ESE)-detected spectrum. The observer frequency ν_obs_ was set to ν_pump_ + Δν with Δν being in the range of 65 MHz and therefore coinciding with the low field local maximum of the nitroxide ESE spectrum. The observer pulse lengths for each DEER experiment were set to 32 ns for both π/2– and π–pulses (with varying intensity) and the pump pulse length was 12 ns.

#### EPR Data Analysis

All EPR data have been evaluated in MATLAB 2021b. Spectral simulations of spin probed HSA samples for temperature denaturation and Scatchard plots were conducted with the MATLAB-based Easyspin 6.0.0 software package.^50^ All corresponding MATLAB codes have been optimized for 2- to 4-component nitroxide spectra. Individual subspectra were double-integrated to extract the spectral fraction concentrations [L]_*t*_·ϕ_*i,j*_ ([L]_*t*_ = total ligand (SLFA) concentration, ϕ_*i,j*_ = temperature-dependent spectral fraction) of corresponding dynamic populations for subsequent thermodynamic analysis. For an appropriate starter set of simulation parameters and the simulation approach the reader is referred to Table S1. All spectral simulations can be found in Figures S3 and S4 in the SI. Fit parameters that were obtained from temperature-dependent Scatchard plots are presented in Tables S2 and S3. Scatchard plots were analyzed according to strategies presented in^8,28,38^ using linear regressions as well as the Rosenthal method.^51^ Temperature-dependent and ligand loading-dependent ln* K*_IC*,j*_ values were also obtained in the course of Scatchard plot evaluation. These curves were reconstructed with exponential fit curves. The cooperativity test for both Scatchard plots (see Figure S5) was conducted as described in refs (8 and 28).

Thermodynamic analyses from CW EPR spectral simulations in this study are largely adopted from the strategies described in^39^ with several adjustments. All physical quantities that emerge from equilibrium constants *K*_A,*j*_ and K_IC,*j*_ have been calculated with fit parameters that were obtained from a sigmoidal fit curve ln *K*_A*,j*_ in eq 1 and a homewritten combination of exponential and double sigmoidal Boltzmann curve regression (ln *K*_IC,*j*_ in eq 3) in Microcal Origin (see Table S4). Fit parameters from the aforementioned curve regressions in Origin were incorporated in the homewritten MATLAB codes that generate appropriate energy plots (Table S5). All corresponding thermodynamic functions for ln* K*_IC,*j*_, Δ*G*°_IC,*j*_, Δ*H*°_IC,*j*_, Δ*S*°_IC,*j*_ and Δ*C*°_P,IC,*j*_ in this graph were computed in a quasi-continuous 500 point grid corresponding to about 0.19 K temperature resolution. Derivations of the expressions given in eqs 5–8 are explicitly shown in the Supporting Information. Characteristic temperatures as *T*_AH,*i*_, *T*_AD,*i*_ and T_H,*i*_ were obtained from relations given in^39^ and are shown in Figure S6. Due to the complexity in Δ*C*°_P,IC,*j*_, the apolar hydration temperature was introduced in analogy to *T*_AD,*i*_, at zero-crossings with increasing temperature.

The raw DEER time domain data as shown in Figure S7 were processed with the MATLAB-based program package DeerAnalysis2019.^52^ For the DEER-derived 16-DSA loading study, all background dimensionalities were set to *D* = 3.74 throughout. These data sets serve as a reference for calculations of the average number of spins from the distribution shape (*N*_*P*(*r*)_). The derivation of an empiric equation that enables to pursue this strategy is based on an exponential fit curve to ln*P*_AB_(*r*); these data are given in the Supporting Information (eq S41). As no spin dilution was applied to these samples, a Langmuir power law could be used for reproducing the modulation depths (Δ) best when 16-DSA loading to HSA varies in the range from about 0.8–6.2 equiv. Similarly, an empirical relation was derived in the Supporting Information that facilitates prediction of the average number of coupled spins based on the modulation depth at room temperature (*N*_Δ_, see eq 11). Due to the change in shape and compactness of HSA with temperature, this approach is preliminary discouraged as the modulation depth experiences additional modification in this respect. All DEER time traces that were obtained from temperature-incubated samples were background-corrected with adjustable spin distribution dimensionalities ranging from 2.00 < *D* < 3.76. These dimensionalities were obtained in a comparative iterative global analysis of all data sets prior to the final Tikhonov regularization procedure. All first moments ⟨*r*⟩ of individual distance distributions *P*(*r*) were plainly obtained from the DeerAnalysis result files.

### DLS Measurements

All DLS data were obtained with an ALV-NIBS high performance particle sizer (HPPS) equipped with an ALV-5000/EPP Multiple Tau Digital Correlator (ALV-Laser Vertriebsgesellschaft m. b. H.). The ALV-NIBS device facilitates HeNe-LASER irradiation with a typical wavelength of λ = 632.8 nm and a 3 mW output power source. Count rates were recorded in a backscattering detection angle of 173° relative to the incident monochromatic light. The sample cell temperatures were adjusted in the range of 9 < *T* < 89 °C in steps of Δ*T* = 4 K by a Peltier temperature control unit. HSA temperature denaturation was conducted on an individual sample in 1.5 mL PMMA semimicro cuvettes (BRAND). For sustaining comparability, a nominal 1-to-1 ratio of 16-DSA was used to equip the protein with the same FA content as in respective CW EPR experiments, well below the CMC = (0.285 ± 0.029) mM. A sample volume of 0.4–0.6 mL was filtered through Rotilabo cellulose acetate (CA) syringe filters with a pore size of 0.2 μM (Rotilabo, Carl Roth GmbH + Co. KG) in order to minimize sample adhesion and therefore protein concentration loss.

**Figure 3:**
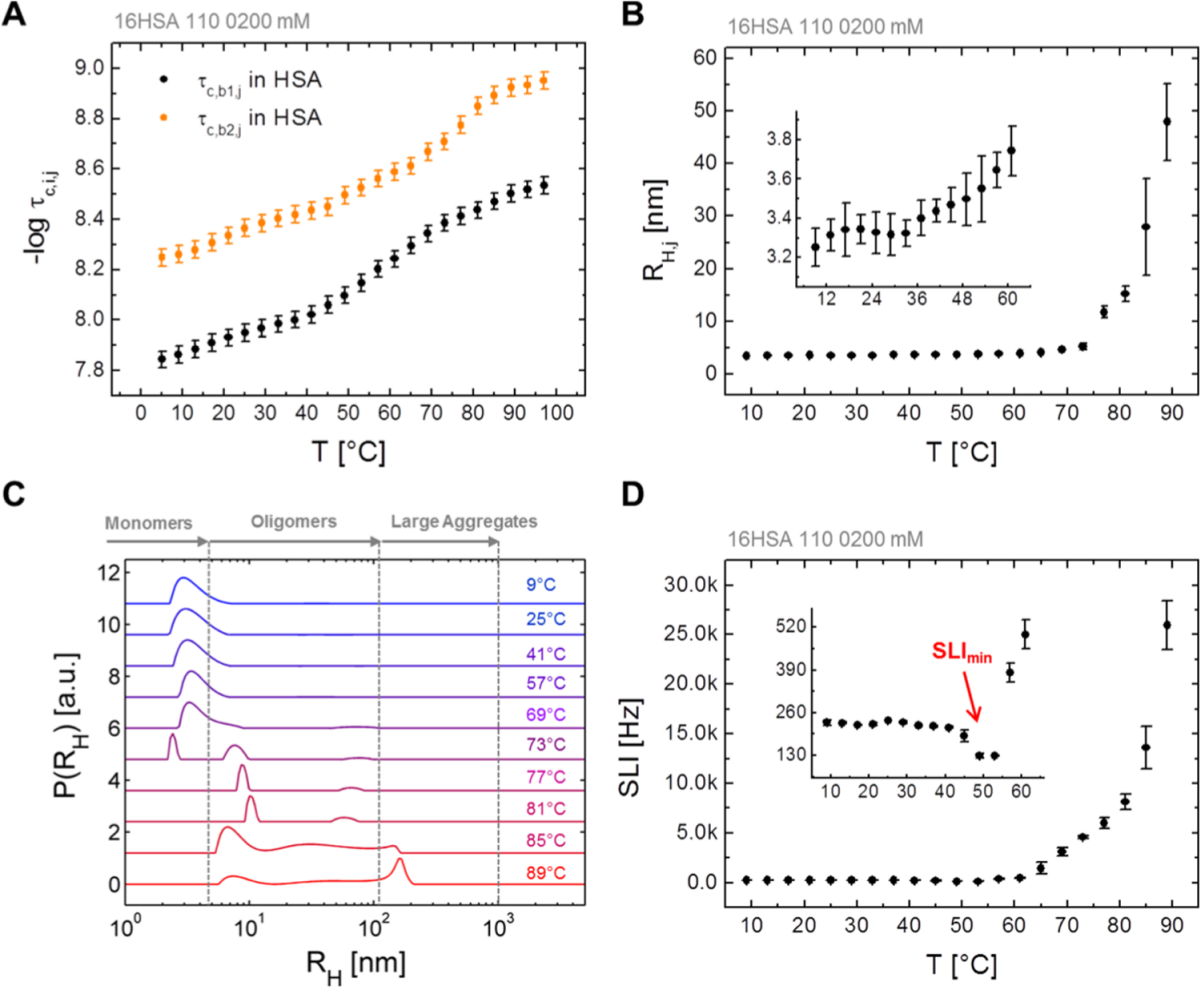
Temperature-induced changes in spin probe and protein dynamics. Comparison of CW EPR and DLS data from a 16-DSA-probed HSA solution in the temperature range from 5 to 97 °C (9 to 89 °C for DLS data) in steps of Δ*T* = 4 K. (A) Semilogarithmic plot of the temperature-dependent rotational correlation times τ_c,*i*,*j*_ from CW EPR of spectral components *b*_1_ (black) and *b*_2_ (orange) of 16-DSA interacting with HSA. Error margins were estimated from spectral simulations to be about 8%. (B) The temperature-dependence of the main hydrodynamic radii *R*_H_ is shown as detected in DLS experiments. Error bars are given as the fluctuation about the mean values of individual measurements at a constant temperature. (C) The temperature-dependent particle size distributions *P*(*R*_H_) in spin probed HSA solutions are given here. Three different regimes are highlighted comprising monomers from 9 to 57 °C, oligomers above 70 °C and larger aggregates above 80 °C. (D) The scattered light intensity (SLI) is given as a count rate (in Hz) from temperature-dependent DLS experiments on 16-DSA-probed HSA with a clear minimum at temperatures between 45 and 57 °C (SLI_min_). All experiments were performed at pH 7.4 with 1:1 nominal equivalents of 16-DSA to HSA at 0.2 mM.

HSA particle size data were extracted from the intensity correlation functions by a *g*_2_(*t*)-DLS exponential and a mass weighted regularized fit in the ALV-NIBS software v.3.0 utilizing the CONTIN algorithm. The refractive index was assumed to be constant at *n*_H2O_ = 1.332 for all temperatures (and λ = 632.8 nm), the water (DPBS buffer) viscosity was corrected for each applied temperature assuming that η_0_ = 1.002 mPa·s at *T* = 20 °C. Deviations in true viscosities from calculated values that are induced by the intrinsic viscosity of HSA are considered as marginal and do not exceed +7.7% at *c*_HSA_ = 0.18 mM = 12.0 mg/mL within 5 °C < *T* < 45 °C. Each sample was measured at least four times at the same temperature for 30 s and was averaged at least over three individual values. The mean values *R*_H,*j*_ of the most prominent size peaks and their statistical fluctuations are given as the standard deviation as depicted in the error bars in Figure 3. The duration of the whole heating procedure was about 8–9 h.

### Differential Scanning Calorimetry (DSC) Measurements

All DSC experiments and data evaluations were performed using a Microcal VP-DSC device (MicroCal Inc.). In all experiments a heating rate of 0.25 K/min was used. Data were recorded with a time resolution of 4 s in the temperature range of 5–95 °C that covers the decisive regions of the corresponding EPR experiments. Two consecutive up and down scans were performed for each sample. However, due to irreversible gelation of the HSA-containing samples at high temperature, only the first thermogram is presented and evaluated. In scan 2–4 only residual heats could be detected and data are therefore not shown.

All 16-DSA-probed HSA solutions were loaded to the sample cell and for comparability the HSA concentration was again set to *c*_HSA_ = 0.18 mM. The ligand-to-protein ratio was varied in the range from about 0–8 equiv, so that altogether eight thermograms were obtained. Pure, degassed DPBS buffer was loaded into the reference cell. Therefore, from all presented thermograms of 16-DSA-probed HSA samples a buffer/buffer reference, as well as a thermogram of pure 16-DSA was subtracted before normalizing Δ*C*_P,HSA_ to the HSA concentrations. Afterward, a cubic baseline was subtracted manually. Data processing was performed with the DSC module for Origin software (MicroCal Inc.). The obtained thermograms were fitted with two Gaussian curves in order to deconvolute both overlapping transitions (*T*_D,1_ and *T*_D,2_). The midpoints of the obtained Gaussians were finally used as transitions temperatures for the two HSA species and are given in the scheme according to Shrake and Ross.^53,54^ A brief rationalization of these results is given in the Supporting Information.

### General Remarks

For simplicity temperature dependent variables will have a *j* as index representing the temperature as *j* = *T* [°C], *i* denotes the dynamic regime (*b*_1_, *b*_2_, *f*, *g*) or any running number, *k* when different samples were considered and *p* to differentiate different phases in a Scatchard plot. All other parameters and indices are explained in the appropriate sections of the main text, the Supporting Information, or in Reichenwallner et al.^39^ The abbreviation “16HSA 110 0200 mM” stands for a standard sample that is composed of 16-DSA-probed HSA in a ratio of 1:1 equiv of 0.2 mM.

## Results and Discussion

### Temperature-Dependent Dynamic Regimes of 16-DSA in HSA Solutions

The temperature dependence of FA binding to HSA, as seen from the FAs’ points of view, generates CW EPR spectra that consist of a superposition of different spectral species (Figure S2 and Table S1 in the Supporting Information). Classical empirical approaches in EPR spectroscopy, such as measuring line amplitude ratios^10^ may not fully capture the complexity of thermal denaturation processes. Therefore, we here use an iterative, manual and global simulation procedure as already established for amphiphilic core–shell polymers.^8,38,39^

EPR spectra of 16-DSA in HSA solutions were investigated in the temperature range from 5 to 97 °C. As known from earlier studies, several dynamic regimes of 16-DSA emerge when this ligand is co-dissolved with HSA. The applied FA loading ratio of 1.13:1.00 at an HSA concentration of 0.18 mM was chosen to be below the critical micellar concentration (CMC) of 16-DSA at all investigated temperatures (*c*_16-DSA_ < CMC = 0.285 mM, see Supporting Information S2). This generally simplifies spectral simulation procedures, although still up to four different subspectra can be identified (Figure 2), however, without micelle-based spectral contributions.

Exemplary, simulation parameters for spectra at three characteristic temperatures (25, 37, and 97 °C) are given in Table S1 and all simulations in the whole temperature range are depicted in Figure S3

16-DSA in HSA solutions at ambient temperatures exhibits two immobilized fractions *b*_1_ and *b*_2_, similar to what can be observed for hydrophobic core – hydrophilic shell polymers in ref (38). A complete set of recorded spectra and all spectral fractions of the simulated species in the full temperature range are presented in Figure 2, where *b*_1_ appears to be the stronger immobilized spectral component while *b*_2_ shows slightly higher dynamics.

Above ∼50 °C, additional free (*f*) and hydrogel-like fractions (*g*)^7,43,55^ are observable, leading to significantly more complex spectra at higher temperatures. The combined *b*_1_ and *b*_2_ fractions comprise 100% of HSA spectra below 50 °C and always exceed 90% of the spectral fraction (ϕ > 0.9) above this temperature. These two spectral components exhibit a highly non-trivial temperature development above 50 °C that will be explicitly discussed below.

The *b*_1_ fraction remains above and *b*_2_ below 50% at all temperatures. The characteristic *a*_iso_ value as a measure of the environmental polarity for *b*_1_ and *b*_2_ is 42.93 MHz for 25 °C as it was also found by Ge et al. for BSA^17^ and decreases to 42.33 MHz (Table S1) at 97 °C. The free (*f*) fraction starts appearing at 53 °C and shows a sigmoidal increase to about 7.0% at 97 °C. The very hydrophobic, hydrogel-like spectral component *g* with an *a*_iso_ value of 40.20 MHz is found above 50 °C but only becomes directly discernible well above 70 °C and reaches a maximum value of 2.1% at 97 °C. It is known that HSA forms ordered macroscopic gel-structures by fibrillation at defined temperatures, pH, osmolality and protein concentrations giving fibrils of 15–30 nm in diameter and 0.1–2.0 μm in length.^56^ We recently reported a thorough physicochemical characterization of the formation of these gel-states from aqueous solutions of HSA and BSA^7^ and have further characterized^7,55^ and developed these gel-state properties since then (see, e.g., ref (57)). This gel-state was also found in amphiphilic core–shell polymers.^38^

When plotting the temperature-dependent τ_c_ values in a semilogarithmic fashion, the −log τ_c_ curves for spectral components *b*_1_ and *b*_2_ both exhibit a sigmoidal shape (Figure 3A). The rotational correlation time ratio τ_c,*b*1_/τ_c,*b*2_ yields a value of 2.46 ± 0.18 when it is averaged across all temperatures, which is very close to 2.65 as proposed for an interconversion from Brownian to free diffusion (*K*_IC_).^38,39,58,59^ While the τ_c_ values decrease from 14.3 ns (5 °C) to 2.9 ns (97 °C) for component *b*_1_, τ_c_-values of *b*_2_ decrease from 5.7 to 1.2 ns in the same temperature range. The most intriguing feature of the −log τ_c_ curves is that τ_c,*b*1_ shows a strong decrease (i.e., −log τ_c_ increase) above 50 °C, while τ_c,*b*2_ only significantly decreases above 70 °C. This decrease in τ_c_ is a direct sign of the structural weakening (finally unfolding) of the protein with increasing temperature. Hence, we detect unfolding by a decrease in ligand immobilization. As established in ref (39), the hydrodynamic properties of FAs in HSA solutions from EPR spectroscopy are now discussed in the light of temperature-dependent DLS experiments (Figure 3B–D).

Structural unfolding of HSA was monitored by plotting hydrodynamic radii *R*_H_ vs temperature (Figure 3B). While the average particle size in the HSA-16-DSA solutions does not change significantly up to 70 °C, a significant size increase takes place above 75 °C. This is due to the emergence of self-association of HSA leading to oligomers of about 50–100 nm above 57 °C and even larger aggregates above 81 °C (Figure 3C, *R*_H_ > 100 nm). Macroscopically, it could be confirmed that the investigated HSA solution indeed forms a gel, as the solutions in the DLS cuvette were completely solidified after the experiments.^7^

Closer examination of the data in the temperature range 9 to 63 °C (see inset in Figure 3B) reveals that the particle size is largely constant at about 3.3 nm in the low temperature range (<40 °C). However, above 45 °C a rather significant particle size increase takes place, still without any larger aggregates appearing. The scattered light intensity (SLI, Figure 3D) was recorded simultaneously and, surprisingly, an identifiable drop in SLI is detected between 45 and 57 °C (SLI_min_) that can be associated with the dissolution of polymer aggregates in our related study on synthetic amphiphilic core–shell polymers.^38^ Prior albumin studies attributed this effect to a mere opening of the Cys34 crevice,^24^ some reversible conformational changes,^60^ or a change in compressibility.^61^ According to Curry et al., the three globular albumin domains (I–III) rotate as rigid bodies relative to one another and fatty acids may stitch these domains together like lock pins.^62^ This dynamic feature may also be modified by temperature.

As expected, the SLI significantly increases again above 60 °C, when also an increasing number of larger oligomeric HSA aggregates form. Taken together, these data indicate that HSA undergoes a structural rearrangement from a tight to a loose organization of subdomains at intermediate temperatures (40–60 °C) that finally leads to protein aggregation by mutual domain or chain entanglement. Technically, this drop in SLI around 50 °C can be understood in terms of an opening of globular HSA molecules. In turn, the decrease of τ_c_ values of the spectral component *b*_1_ (Figure 3A) monitoring the global Brownian diffusion confirm the picture of a structural softening of HSA at about 40–50 °C. The τ_c_ values of the free diffusion process as monitored by *b*_2_ do not exhibit this decrease to such an extent. This circumstance indicates that the domains harboring the 16-DSA ligand remain largely unaffected up to about 70 °C. All these DLS and EPR data corroborate a temperature-induced rotational decoupling of the three HSA domains, reminiscent of an entangled three-piece *boleadora*, a Pre-Columbian South American throwing weapon with weights at the ends of three interconnected cords that is used to capture animals by entangling their legs.

### Temperature-Dependent Ligand Uptake Capabilities of HSA

**Figure 4:**
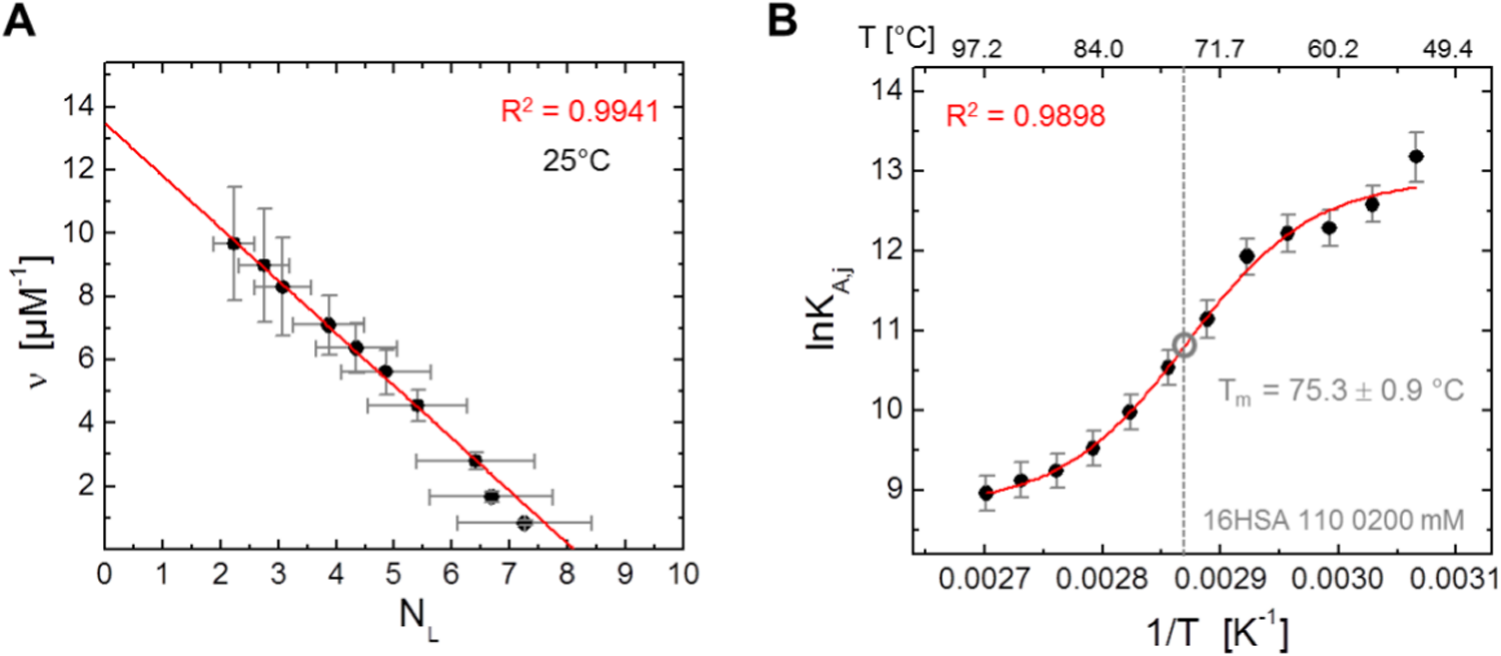
16-DSA binding affinity and capacity of HSA. (A) Scatchard plot of 16-DSA interacting with HSA at 25 °C (black, full circles) with linear fit (red). The Scatchard plot was constructed in the loading ratios from 2:1 to 8:1. (B) van’t Hoff plot of the association constant *K*_A,*j*_ is shown that can be constructed from free (*f*) and bound (*b*_1_, *b*_2_, *g*) spectral fractions in Figure 2C for *T* ≥ 53 °C. A sigmoidal Boltzmann fit curve is shown together with the midpoint temperature *T*_m_ (=α_3_^–1^, see eq 1). The ligand-to-protein ratio for the van’t Hoff plot is 1.13:1.00 at 0.18 mM equivalents. The quality (*R*^2^) of respective fit curves is very high and given as red insets.

The 16-DSA binding capacity has been determined for HSA via a Scatchard plot analysis^63^ after simulation of CW EPR spectra. The simulated CW EPR spectra can be found in the Supporting Information (Figure S4). Two different temperatures have been chosen to investigate the effect that temperature has on the ligand binding properties, one at room temperature (25 °C) and another one at physiological temperature (37 °C). Both Scatchard plots were constructed for ligand/protein loading ratios from 2:1 to 8:1. At 25 °C a linear plot allows for extracting the number of equivalent binding sites *N*_E_ and the association (see eq S5, *K*_A_) or dissociation constant (*K*_D_, see Figure 4A). The Scatchard plot at 25 °C reveals a total number of *N*_T,25_ = 8.1 ± 0.3 equiv binding sites of HSA for 16-DSA with a dissociation constant of *K*_D_ = (603 ± 16) nM, corresponding to an association constant of *K*_A_ = (1.66 ± 0.04) × 10^6^ M^–1^ (see Table 1). However, at 37 °C linearity in the Scatchard plot vanishes and an exponentially decaying curve shape can be observed (Figure S5A). The Rosenthal method^51^ was applied revealing a slight increase in the total number of binding sites (*N*_T,37_ = 9.2 ± 2.9). The binding sites can be subdivided into two groups of either strong (*N*_E,37,I_ = 4.0 ± 1.3) or weak binding sites (*N*_E,37,II_ = 5.2 ± 1.6). This concept was already described by Karush^64^ for exemplifying cooperativity of ligand binding to albumins which describes the observed processes best. It can be rationalized that the total number of binding sites might slightly change with temperature.

**Table 1:** Thermodynamic Data from 16-DSA Binding to HSA

*T* [°C]	*N*_T,*j*_	*p*	*N*_E_,_*j*_,_*p*_	*K*_A_,_*j*_,_*p*_ [M^–1^]	*K*_D_,_*j*_,_*p*_ [M]	Δ*G*°_A*,j,p*_^d^ [kJ/mol]	*C*_*j*_,_*p*_^e^
25^a^	8.1 ± 0.3			(1.66 ± 0.04) × 10^6^	(6.03 ± 0.16) × 10^–7^	–35.5 ± 0.9	(N.C and – )
37^b^	9.2 ± 2.9	I	4.0 ± 1.3	(5.40 ± 0.74) × 10^6^	(1.85 ± 0.25) × 10^–7^	–40.0 ± 5.5	(−)
		II	5.2 ± 1.6	(2.50 ± 0.60) × 10^5^	(4.00 ± 1.01) × 10^–6^	–32.0 ± 7.7	(− and + )
97^c^				(7.84 ± 0.49) × 10^3^	(1.27 ± 0.03) × 10^–4^	–27.6 ± 8.3	N.A.

a Values are obtained from the Scatchard plot in Figure 4A.

b Values are obtained from the Rosenthal analysis that was applied to the Scatchard plot in Figure S5A, here with two Scatchard phases *p*.^8^

c Values are obtained from the van’t Hoff plot of ln* K*_A_ in Figure 5B.

d Molar Gibb’s free energy change of ligand association at a given temperature and Scatchard phase *p*.

e Temperature-dependent ligand binding cooperativity of Scatchard phases *p*.

A cooperativity test can be performed according to Tanford^1^ that reveals an interesting feature when both Scatchard plots are compared in terms of ln* K*_A,int_* (Figure S5B, see refs (8,28) for details on the derivation). In principle, the plot of ln* K*_A,int_* versus *N*_L_ yields the concentration-dependent, stepwise energetic contributions of associated ligands. The interpretation is made according to the suggestion of De Meyts and Roth.^65^ At 25 °C, the typical noncooperative (N.C.) region is identified up to *N*_L_ ≈ 6.5, as it was similarly observed for unmodified BSA that is followed by negative cooperativity up to *N*_L_ = 8. However, at 37 °C, there is a region of negative cooperativity for *N*_L_ < 6.5, which switches to positive cooperativity above this value. Although the altered behavior at 37 °C might imply activated physiological function, for simplicity and a more precise determination of the total number of binding sites (*N*_T_), CW EPR data at 25 °C may be better suited for standard analyses.

Additionally, a van’t Hoff plot of the temperature-dependent association constant *K*_A_ can be constructed from data shown in Figure 2C (1.13 equiv of 16-DSA per HSA). In this case we showed that the tight binding regime is valid ([*L*]_*t*_ < [*R*]_*t*_ = *N*_T_·*c*_HSA_).^39^ For simplicity, the total receptor concentration in the solution is assumed to remain constant at a value of [*R*]_t_ = 1.46 mM for all temperatures, as it is only of interest where the strongest change in affinity occurs. Additionally, the bound fraction is here merely derived from the sum of spectral fractions ϕ of dynamic regimes *b*_1_, *b*_2_ and *g* (ϕ_*b*_ = ϕ_*b*1_ + ϕ_*b*2_ + ϕ_*g*_).

As 16-DSA binding at lower temperatures is so strong that the free (*f*) fractions are indiscernible from noise (ϕ_*f*,5–49_ < 0.02%) at the start loading ratio used here, a temperature dependent van’t Hoff plot of *K*_A_ in this temperature range is unfortunately inaccessible (5 °C – 53 °C). However, a fit function can be applied to the ln* K*_A,*j*_ curve above 53 °C as shown in Figure 4B to obtain a better quantitative description of the temperature-dependent ligand binding process. The general shape of ln* K*_A,*j*_ is best described by a Boltzmann function of the kind
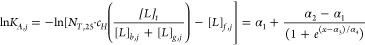
1where α_*z*_ (*z* = 1–4) are fit parameters that are given in Table S4. For mathematical clarity, the inverse temperature from the van’t Hoff plots is now replaced by *x* = *T*^–1^. This description of the ligand association constant *K*_A_ contains the initial assumption that HSA denaturation is a hypothetical two-state phase transition, e.g., from a solid (s) to a vapor (v) state.^66^ In literature, nonlinear van’t Hoff plots in general were already reported for other systems, too.^67,68^ An assignment of physical meaning to the fit parameters in eq 1 can be developed in the following way: the denaturation process of HSA occurs in a defined temperature range Δ*T* with a midpoint temperature *T*_m_ = α_3_^–1^ between the states of energy *H*_1_ = α_1_ and *H*_2_ = α_2_.^69^ The respective temperature range Δ*T* = (α_3_ – α_4_)^−1^ – α_3_^–1^ = 6.61 K of the transition also defines the corresponding slope *k*_*K*A_ = (α_2_ – α_1_)/(4Δ*T*) = 0.155 K^–1^ at *T*_m_.^69,70^ The midpoint temperature or melting temperature of HSA is therefore at *T*_m_ = 75.3 ± 0.9 °C, as determined from the maximum change in ligand binding affinity. Table 1 reveals that the most affected functional property of HSA upon temperature increase is the decrease of the macroscopic ligand association constant *K*_A_ by more than 2 orders of magnitude (∼200 times, assuming that *N*_T_ = *N*_T,25_ = 8.1 at all temperatures). Therefore, the free energy of 16-DSA association also becomes less negative and the observed ligand binding process thus becomes less exergonic (Δ*G*°_A,97_ – Δ*G*°_A,25_ = 7.9 kJ mol^–1^).

These data are in very good agreement with results obtained by Gantchev and Shopova,^16^ who also reported *N*_T_ = 8 ± 1 for HSA. Furthermore, almost identical free energies were found for palmitic acid binding to HSA. Spector et al.^71^ also found two classes of binding sites with Δ*G*°_A,37,I_ = −39.8 kJ/mol and Δ*G*°_A,37,II_ = −32.2 kJ/mol. An ITC study by Aki and Yamamoto revealed similar results for palmitic acid interacting with HSA (Δ*G*°_A,37_ = −38.8 kJ/mol).^72^

### Thermodynamic Analysis of the Interconversion Process of 16-DSA Bound to HSA

After the first EPR-based analysis of FA binding to HSA, now the temperature-dependent interconversion process before and during the denaturation process of HSA is characterized from the bound ligands’ point of view and the emerging immobilized spectral fractions (ϕ_*bi*_, see^39^ for further details). Values for ln* K*_IC_ are constructed from Figure 2B according to eq 2 (*K*_IC_ = ϕ_*b*2_/ϕ_*b*1_) and the corresponding temperature response of the system consisting of 16-DSA bound to HSA is shown in Figure 5.
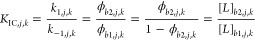
2

**Figure 5:**
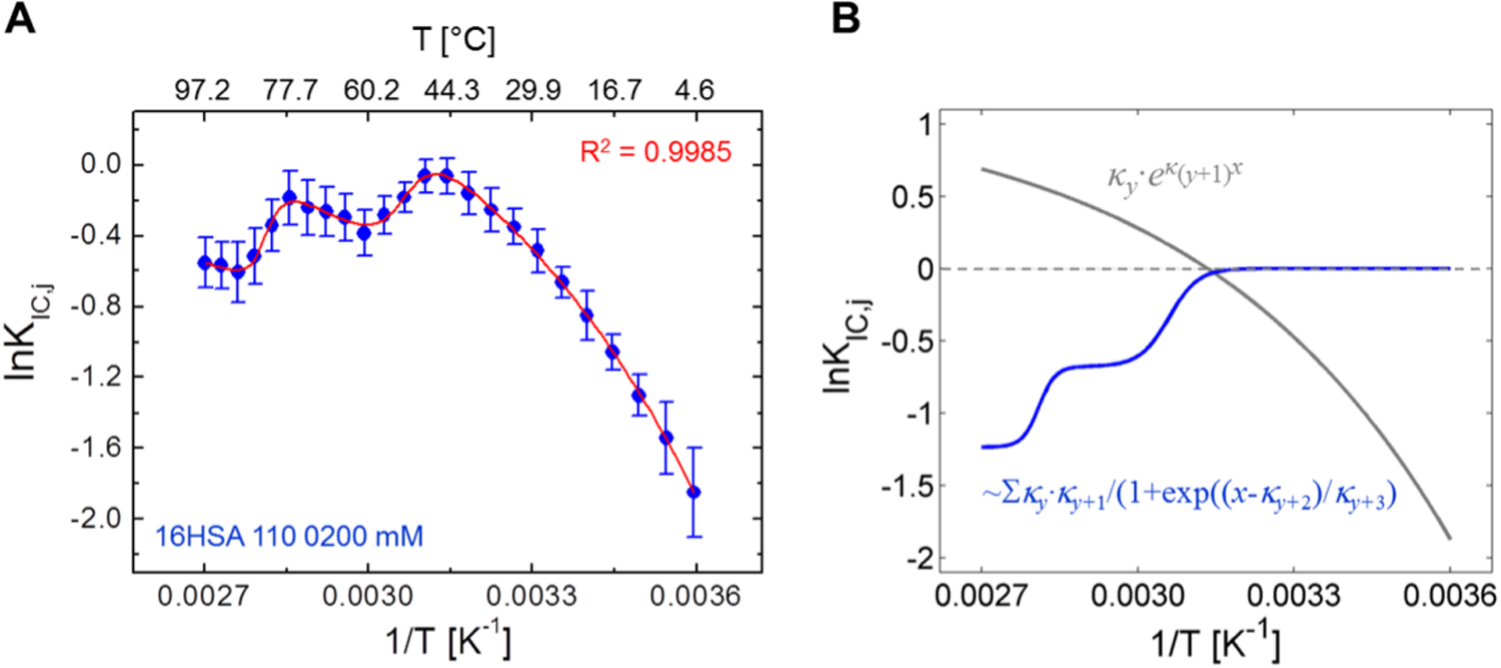
A van’t Hoff plot of ln*** K***_**IC**_ from 16-DSA bound to HSA. (A) Individual data points of the van’t Hoff plot for ln* K*_IC_ were calculated from eq 2 with the spectral fractions ϕ_*b*1_ and ϕ_*b*2_ of 16-DSA bound to HSA (blue) in the temperature range from 5 to 97 °C. A fit curve derived from eq 3 is shown in red (*R*^2^ = 0.9985). (B) Mathematical decomposition and presentation of individual components of the fit function for ln* K*_IC,*j*_ highlighting the second and third term of eq 3.

As shown for core–shell polymers, an arbitrary fit function with the only purpose to reproduce the curve mathematically can been found for the van’t Hoff plot of ln* K*_IC_ that is given in eq 3 (*x* = *T*^–1^). The sum of the exponential second term of eq 3 and a double Boltzmann expression (the complete third term of eq 3 in parentheses) mathematically fits best to describe the temperature-dependent progression of the interconversion equilibrium
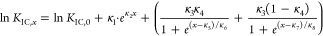
3yielding a correlation coefficient of *R*^2^ = 0.9985. Pure polynomial functions fully fail in reproducing this ln* K*_IC_ curve shape. All corresponding fit parameter values of ln *K*_IC,0_ and κ_*y*_ (*y* = 1 – 8) are shown in Table S5. Optionally, the second term κ_1_·exp(κ_2_·*x*) can also be written as a polynomial equation *u*·*x* + *v*·*x*^2^. The resulting fit curve of ln* K*_IC,*j*_ and the rationale for separating individual contributions is given in Figure 5. The application of a double Boltzmann term generally implies that two dynamic transitions may occur in HSA that affect the temperature course of ln* K*_IC,*j*_.

The parameters κ_3_ and κ_4_ can be understood as arbitrary step heights of the transitions and parameters κ_5_ and κ_7_ are the midpoint temperatures *T*_m_ of the individual transitions, while κ_6_ and κ_8_ are the individual transition widths. A straightforward thermodynamic analysis can be conducted for ln* K*_IC_ of 16-DSA interacting with HSA in accordance to the strategy developed in^39^ (eqs S8–S34 in the Supporting Information).

The temperature-dependent interconversion enthalpy change is obtained from eq 3 with the relation
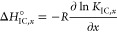
4resulting in
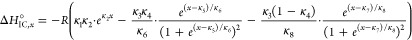
5

Together with the relation Δ*G*°_IC_ = Δ*H*°_IC_ – *T*Δ*S*°_IC_ = –*RT* ln* K*_IC_, at equilibrium the temperature-dependent molar interconversion entropy change may also be determined as
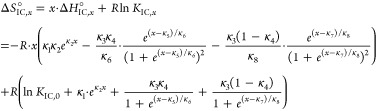
6

As described in the Supporting Information, the differential equation of the molar heat capacity change Δ*C*°_P,IC_ = dΔ*H*°_IC_ /d*T* is best calculated by
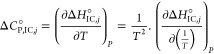
7a
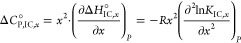
7byielding
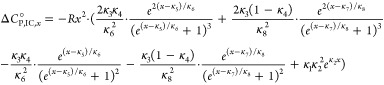
8

**Figure 6:**
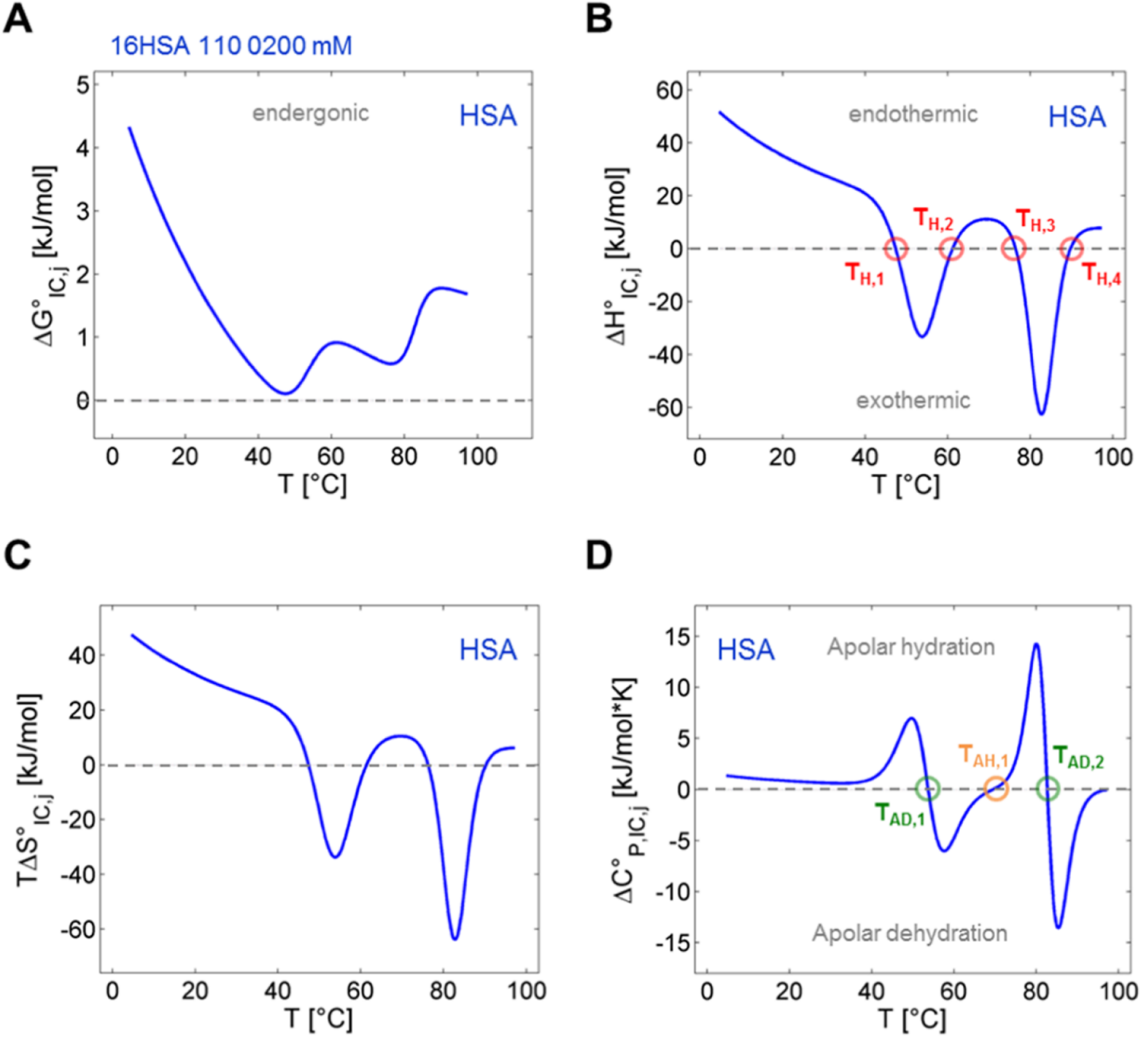
Graphical representation of the thermodynamic functions derived from ln* K*_IC,*j*_ with 16-DSA bound to HSA. Continuous depiction of (A) molar Gibb’s free energy change Δ*G*°_IC,*j*_ that remains endergonic in the whole temperature range. (B) Temperature-induced change of molar enthalpy Δ*H*°_IC,*j*_ calculated from eq 5 comprising endothermic and exothermic regions and enthalpy compensation temperatures *T*_H,1_ – *T*_H,4_ highlighted with red circles. (C) Temperature-dependent change in molar entropy *T*Δ*S*°_IC,*j*_ calculated from eq 6. (D) Change in molar heat capacity Δ*C*°_P,IC,*j*_ with apolar dehydration temperatures *T*_AD,1_ and *T*_AD,2_ in green and the apolar hydration temperature *T*_AH,1_ in orange. The corresponding curve was calculated from eq 8. All curves are obtained from the interconversion process ln* K*_IC,*j*_ from data presented in Figures 2B and 5A.

Explicit derivations are given in the Supporting Information S6. In Figure 6 all functions are plotted in the full temperature range. Note that the spectral fraction of *b*_1_ does not decrease below ϕ_*b*1,*j*_ = 0.5, thus leading to constantly negative ln* K*_IC_ values (Figure 6A). This means that Δ*G*°_IC_ is always endergonic and the interconversion process is therefore unfavorable throughout the whole observed temperature range. However, the Δ*G*°_IC_ curve exhibits a global minimum at 47.4 °C, where the free energy approaches zero (Δ*G*°_IC_ ≈ 0.11 kJ/mol). Intriguingly, this is the temperature region in which also the onset of the HSA expansion, or structural opening, can be observed in the corresponding DLS data of Figure 3D (SLI_min_). Above 47.4 °C the interconversion process of bound 16-DSA is again more unfavorable.

Unlike Gibb’s free energy, the corresponding enthalpy changes Δ*H*°_IC_ of the interconversion process (Figure 6B) strongly alternate between endothermic and exothermic. Altogether, four enthalpy compensation temperatures are found, *T*_H,1_ = 47.4 °C, *T*_H,2_ = 61.1 °C, *T*_H,3_ = 76.5 °C, and *T*_H,4_ = 89.7 °C. Hence, this process is entropy driven (Δ*H*°_IC_ > 0, Δ*S*°_IC_ > 0) and endothermic below 47.4 °C, from 61.1 to 76.5 °C, and above 89.7 °C. In the intervals between 47.4 to 61.1 °C and 76.5 to 89.7 °C the dynamic interconversion process of 16-DSA is enthalpy driven (Δ*H*°_IC_ < 0, Δ*S*°_IC_ < 0) and energy is released (exothermic). These characteristic temperatures are summarized in Figure S6 and were calculated according to eqs S35–S37 in the Supporting Information as in.^39^ Molar entropy changes *T*Δ*S*°_IC,*j*_ exhibit very analog behavior (Figure 6C).

The changes in molar heat capacity Δ*C*°_P,IC_ in Figure 6D reveal a slight apolar hydration (Δ*C*°_P,IC_ > 0) below 38 °C, before a significant increase is detected with a maximum at 49.7 °C. This is again indicative of an opening of the HSA structure that exposes some hydrophobic regions containing 16-DSA ligands to the solvent. The zero-crossing at *T*_AD,1_ = 53.6 °C terminates the apolar hydration temperature region. Note that the solvent exposure of hydrophobic regions/ligand coincides with the onset of detectable free ligand fractions ϕ_*f*_ and the gel fraction ϕ_*g*_. This means that protein aggregation is triggered and ligand binding becomes less favorable, as it was already shown for core–shell polymers with intermediate-sized hydrophobic cores.^39^ Accordingly, the structural integrity of HSA is notably deteriorated above *T*_AD,1_.

This loss in protein structure and functionality is again converted into an apolar hydration process above *T*_AH,1_ = 69.4 °C. This (second) apolar hydration process is much more pronounced than the first one (from about 38 to 53 °C) with a maximum appearing at 80.0 °C. This temperature range (70 to 80 °C) also shows the strongest decrease in HSA’s ligand binding affinity (Figure 4B, ln* K*_A_), where the corresponding midpoint temperature is found at *T*_m_ = 75.3 ± 0.9 °C. At *T*_AD,2_ = 82.7 °C, apolar dehydration is re-established and the SLI from DLS experiments exhibits the strongest increase. This final apolar dehydration therefore coincides with the formation of larger HSA aggregates (Figure 3C), ultimately leading to macroscopic gelation

### Comparison of Spectroscopic ln* K*_IC_ with Results from DSC

**Figure 7:**
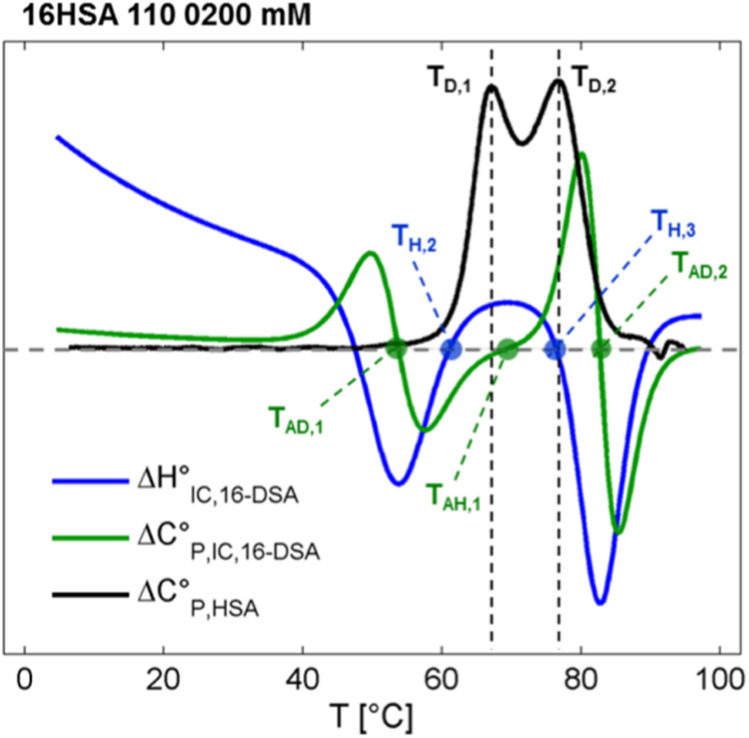
Comparison of ln* K*_IC_ thermodynamics with a DSC thermogram of 16-DSA bound to HSA. Here, an overlap of EPR data together with a DSC thermogram is shown that represents heat capacity changes of HSA molecules (Δ*C*°_P,HSA_, black) comprising denaturation temperatures *T*_D,1_ and *T*_D,2_. EPR data are taken from Figure 6B (Δ*H*°_IC,16-DSA_, blue) and Figure 6D (Δ*C*°_P,IC,16-DSA_, here green) that highlights 16-DSA-based interconversion enthalpy compensation temperatures *T*_H,2_ and *T*_H,3_, as well as changes of ligand hydration at *T*_AD,1_, *T*_AH,1_ and *T*_AD,2_. The exact ligand-to-protein ratio for both methods was 1.1:1.0.

The EPR-derived thermodynamic analyses of the ligand-based parameter ln* K*_IC_ can be compared to the changes in heat capacity of HSA as observed in DSC experiments. Accordingly, this approach combines nanoscopic thermodynamic properties obtained from the SLFAs’ points of view with the macroscopic heat signature of the protein and ligands solution. Thus, samples have been prepared identically, each containing 16-DSA-to-HSA equivalents of 1.1:1.0 at *c*_HSA_ = 0.18 mM. The DSC thermogram in Figure 7 (black) exhibits the classical bimodal appearance of albumin loaded with FAs^53,73^ showing maxima at the two denaturation temperatures *T*_D,1_ = 65.1 °C and *T*_D,2_ = 75.1 °C. This bimodal thermogram was initially interpreted to include a ligand redistribution process during denaturation.^53,54,74^ The FAs released from molten, or denatured HSA molecules were thought to be absorbed by the remaining intact HSA, contributing their binding energy to internal protein stability.^53^ This is a common phenomenon in ligand binding to macromolecules.^75^ Here, our EPR-derived thermodynamic data do not reproduce the DSC thermogram shape but rather reveal some of the underlying dynamic processes as observed from EPR in HSA-ligand interconversion. First of all, the two denaturation temperatures *T*_D,i_ from DSC are not directly found in thermodynamic analyses of ln* K*_IC_.

Fit parameters for midpoint temperatures in Figure 5A are identified as the apolar dehydration temperatures *T*_AD,1_ (κ_5_^–1^) and *T*_AD,2_ (κ_7_^–1^) that represent zero crossings of Δ*C*°_P,IC,*j*_, roughly coinciding with the onset and end of heat capacity changes in HSA as detected in DSC experiments (Δ*C*°_P,HSA_). Consistently, the *T*_AD_ values give the minima of the Δ*H*°_IC_ and Δ*S*°_IC_ curves. However, this direct correlation of DSC and EPR curves reveals that *T*_AH,1_ is close to the minimum between *T*_D,1_ and *T*_D,2_, in the DSC curve, while *T*_H,3_ almost exactly coincides with the second melting peak (*T*_D,2_). The enthalpy compensation temperature *T*_H,3_ also indicates the strongest increase or maximum slope in apolar hydration as observed from Δ*C*°_P,IC_ of the ligand interconversion process.

On a more fundamental level, the ligand interconversion changes to endothermic reaction conditions for temperatures between *T*_H,2_ and *T*_H,3_, while the first melting peak develops and ceases (*T*_D,1_). This means that the interconversion process of 16-DSA becomes energetically more favorable during the first phase transition in the HSA substrate when also interconversion entropy *T*Δ*S*°_IC_ rises. This behavior suggests that ligand and protein undergo a mutual energetic coupling, at least to a certain extent.

The conclusion can be drawn that apolar dehydration temperatures *T*_AD_ from ligand interconversion coincide with fine-tuned structural rearrangements in albumin that trigger denaturation and aggregation. However, both *T*_AD,1_ and *T*_AD,2_ in HSA differ from the denaturation temperatures (*T*_D_) and are not detected in DSC experiments as they rather confine the temperature range of the whole biphasic denaturation process (53.6–82.7 °C). In contrast, the apolar hydration temperature *T*_AH,1_ marks the onset of a transient structural rearrangement that induces an increased structural stability in HSA, even at high temperatures. From an energetic viewpoint, FAs can be seen to absorb heat in form of a favored interconversion and indicate the second melting peak (*T*_D,2_). The simultaneous increase in interconversion entropy (*T*_AH,1_ = 69.4 °C) illustrates that the ligand obtains additional degrees of motional freedom.

**Figure 8:**
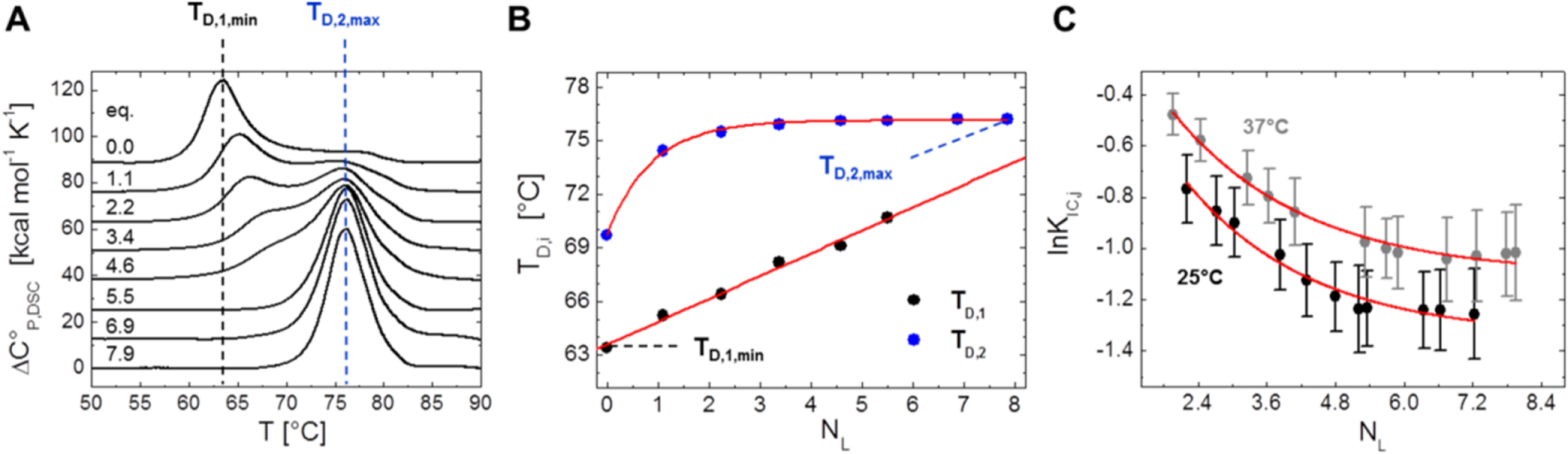
Temperature stability shift of HSA by 16-DSA loading in the view from DSC and EPR. (A) DSC thermograms of HSA loaded in the range from 0 to about 8 FA equivalents. The melting temperatures *T*_D,1,min_ and *T*_D,2,max_ represent the minimum and maximum melting temperatures as induced by the absence or presence of 16-DSA, respectively. (B) Melting temperatures *T*_D,1_ (black) and *T*_D,2_ (blue) of HSA depending on FA loading equivalents *N*_L_. Individual values are taken from thermograms in (A). Linear (eq S38) and exponential (eq S39) fit curves are shown in red with characteristic values *T*_D,1,min_ and *T*_D,2,max_ highlighted. Fit parameters can be found in Table S6. (C) Influence of 16-DSA loading on ln* K*_IC,*j*_ for 25 °C (black) and 37 °C (gray) as derived from EPR spectroscopy. All ln* K*_IC,*j*_ values are taken from simulations that were used for Scatchard plot construction (Figure S4) with respective exponential fit curves according to eq S40 shown in red. Fit parameters can be found in Table S7.

The change in temperature stability upon 16-DSA loading was also investigated by DSC. A typical ligand-induced conversion in the melting behavior (*T*_D_) is seen in Figure 8A ranging from bimodality at lower FA equivalents (up to ca. 5 equiv) to a single melting peak at higher loadings. The relative increase in melting temperatures is depicted in the scheme of Shrake and Ross.^53^ While the first melting peak (*T*_D,1_) is shifted linearly upon 16-DSA loading, the second melting peak position (*T*_D,2_) reaches a plateau value of 75 °C already between 1 and 2 equiv (Figure 8B).

Additionally, the fit functions for *T*_D_ give a lower (*T*_D,1,min_ = 63.6 °C) and upper (*T*_D,2,max_ = 76.1 °C) denaturation temperature limit that defines the maximum observed stability increase of Δ*T*_D,max_ = 12.5 °C as induced by the presence of 16-DSA (Figure 8B). These values are in very good agreement with reported values^76−78^ and—as a side note—also confirm that the commercially available HSA really was largely FA-free.

Simulation data from both Scatchard plots (Figure S4**)** provide a viewpoint on how 16-DSA loading leads to a global shift in the interconversion equilibrium (*K*_IC_). Similar to results that are obtained from *T*_D,2_ in DSC experiments, no further global change in ln* K*_IC_ is observed when HSA is loaded with about *N*_L_ ≥ 5 FAs regardless of temperature (Figure 8C). Intriguingly, not only temperature but also 16-DSA loading defines to which extent the interconversion process is endergonic. While a temperature increase shifts this equilibrium to free diffusion (*b*_2_), an increased 16-DSA loading shifts the ligand interconversion back to Brownian diffusion (*b*_1_), i.e., ln* K*_IC_ generally decreases when the *b*_1_ fraction increases. Therefore, dynamic ligand interconversion gets more unfavorable with higher FA loading. Hence, fatty acid ligands in general can be truly seen as (negative) temperature equivalents for HSA as they contribute their free energy of binding to protein stability, however, only partially.^75,79^ In this regard, the slope ∂*T*_D,1_/∂*N*_L_ from the linear fit to *T*_D,1_ in Figure 8B gives a value of *k*_TD,1_ = 1.28 ± 0.06 °C/FA. Thus, the first HSA melting temperature is shifted for about +1.3 K with each additional 16-DSA molecule. Both exponential decays of ln* K*_IC_ in Figure 8C exhibit an almost identical decay constant *B*_K*,j*_ = 2.2 ± 0.3 FA, indicating independence of temperature. Therefore, temperature (here: Δ*T* = 37 °C – 25 °C = 12 K) mainly shifts the ligand concentration dependent ln* K*_IC_ isotherm along the *y*-axis. Accordingly, the difference Δln* K*_IC,Δ*T*_ is assumed to be largely constant for all 16-DSA loadings. This value lies around 0.24 and corresponds to ΔΔ*G*°_IC_ = 0.48 kJ/mol, as determined by ln* K*_IC,0_ for the plateau at *N*_L_ → ∞ (see also Table S7). We conclude, that FA binding reduces internal flexibility in HSA with a simultaneous gain in temperature stability and indirectly confirms the picture of FAs as “lock pins”.^77^ 16-DSA, or presumably FAs in general, can be seen as natural pharmacological chaperones (“pharmacoperones”), i.e., small molecules that promote refolding of proteins exposed to stress or with dysfunctional mutations.^80,81^

### Further Insights into Ligand Redistribution Using DEER Spectroscopy

In case that the proposed stabilizing ligand redistribution^54^ takes place during temperature-induced HSA denaturation, the binding site occupation of the remaining intact proteins should change to a certain extent. Using DEER spectroscopy, we have established the “fingerprint” distribution of FA binding sites, of the “entry points” to the sites on the protein’s surface as well as to the anchor points (amino acid binding site) in its interior.^3,6,41−43^ Furthermore, we found that for BSA in solution the characteristic FA distributions coincided very well with the ones expected from the crystal structure (of BSA and HSA), while for HSA this is only true for its interior – the entry point distribution of FAs strongly deviates from the crystal structure.^3,6,41,43^ Yet, the entry site distribution in HSA is remarkably simple (less anisotropic) and is essentially bimodal with distance peaks at 2.2 and 3.6 nm.^3,41^ We here use an empirical analysis of the ratio of these two peaks in direct dependence of the solution temperature before shock-freezing the samples. This is necessary for DEER experiments with nitroxide radicals, which have to be carried out at cryogenic temperatures (here: 50 K). Therefore, small amounts of cryoprotectants like glycerol have to be added to guarantee vitrification of the temperature-induced albumin ensemble^3,82^ and associated changes in the subdomains.

### Characterization of Ligand Redistribution Using DEER Spectroscopy

CW EPR results have shown that a strong decrease in binding affinity toward 16-DSA takes place at temperatures above 53 °C, which indicates the release and redistribution process of bound ligands during HSA denaturation but does not allow one to locate the remaining binding sites. In Figure S5 it is furthermore shown that binding site cooperativity changes with temperature and we have tested whether 4-pulse DEER can provide further insights into the redistribution, or interconversion process.

It was already shown in Junk et al.^42^ that HSA exhibits much smaller modulation depths for a 16-DSA loading ratio of 2:1 than derived from model biradicals, despite a similar number of coupled spins should be present. Therefore, standard spin counting procedures that utilize standardized inversion efficiencies λ do not apply properly for this self-assembled system. Another study also emphasizes the effect that cooperativity may have on modulation depth.^83^ Furthermore, the potential number of accessible binding sites in this study (here: *N*_T_ = 8.1 ± 0.3, see Table 1) gives rise to tremendous multispin effects that leads to overestimation of short distances and suppression of large distances. First, the previous experiments from Junk et al.^42^ are here similarly reproduced with the 16-DSA-probed HSA system (here in equivalents of *c*_H__SA_ = 0.18 mM). In an initial step, CW EPR was applied on an aliquot from the final DEER sample in order to determine the true FA concentration in the sample solution by double integration (Figure S7A).

As it was shown in,^84^ all binding pockets are occupied to a certain extent in the solution ensemble of HSA, even at a 1:1 loading ratio. This is indirectly confirmed by the Scatchard plot at 25 °C in Figure 4A giving *N*_T,25_ = 8.1 ± 0.3 equiv, noncooperative binding sites. Even at the highest loading ratios of about 1:7 (*N*_L_ ≈ 7), more than 98.5% of supplied 16-DSA remains bound to HSA.

**Figure 9:**
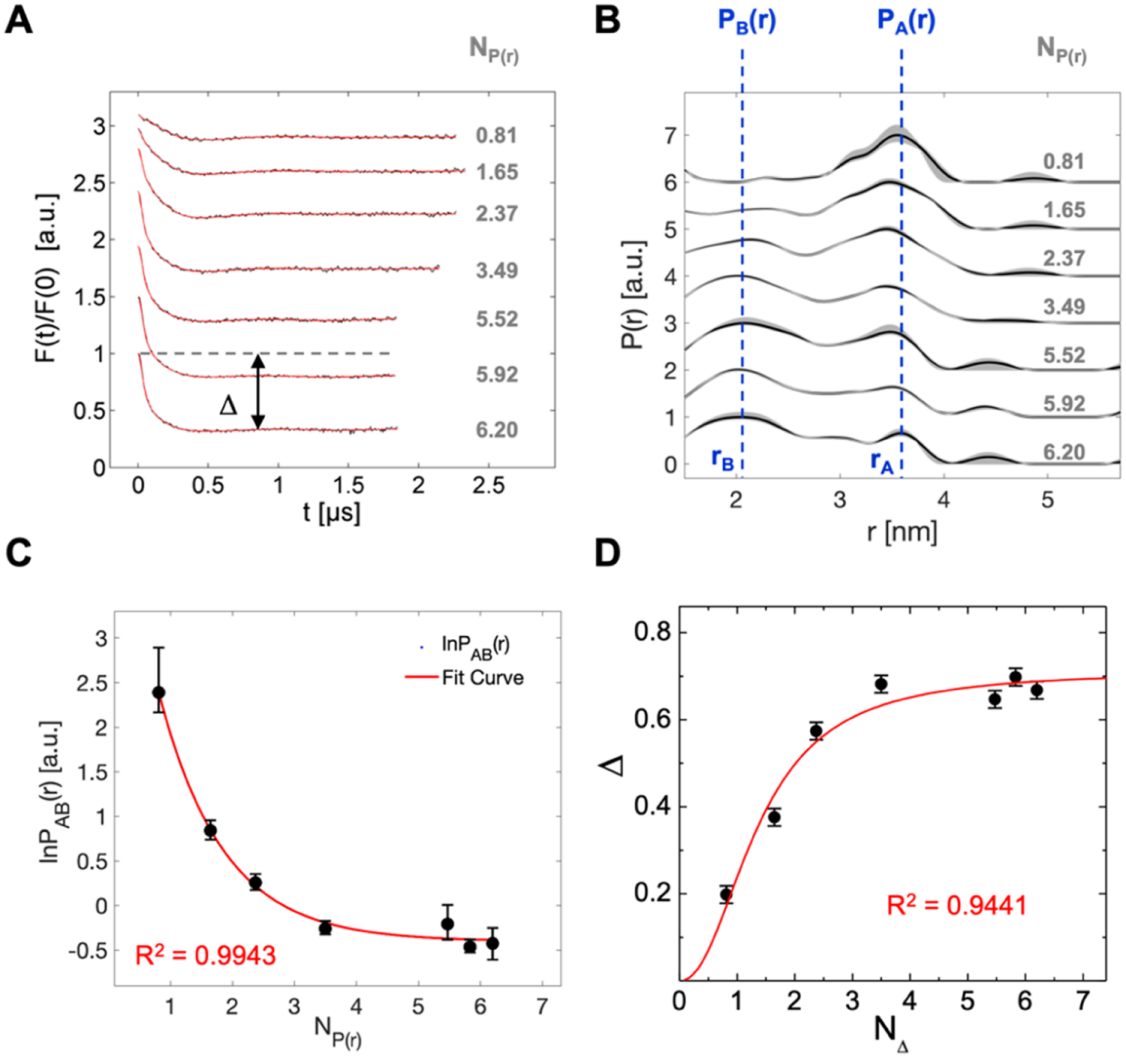
Effect of ligand loading in DEER data for 16-DSA-probed HSA solutions. HSA was loaded with 16-DSA equivalents from 0.81 – 6.20 at *c*_HSA_ = 0.18 mM. (A) Corresponding dipolar evolution functions *F*(*t*)/*F*(0), regularized fits (red), modulation depths Δ (gray dotted line, indicated for highest loading ratio 6.2) and (B) resulting distance distributions *P*(*r*). The two most prominent features in *P*(*r*) are denoted as *P*_A_(*r*) at *r*_A_ = 3.5 ± 0.1 nm and *P*_B_(*r*) at about *r*_B_ = 2.2 ± 0.2 nm. The validated distance distributions *P*(*r*) were determined with DeerAnalysis2019. Confidence intervals of all *P*(*r*) curves are given as gray shaded areas. (C) Data points for ln*P*_AB_(*r*) (black dots) are shown as a function of 16-DSA concentration equivalents *N*_*P*(*r*)_. An exponential fit curve (red) is applied to the ln*P*_AB_(*r*) curve (red) that finally yields eq 9. Asymmetric error bars were extracted as variations from values of *P*_A_(*r*) and *P*_B_(*r*) in *P*(*r*) curves in (B). (D) The modulation depths Δ are presented as a function of 16-DSA equivalents *N*_Δ_ (black dots) with a fit curve (red) corresponding to eq 10. Both fit curves in (C) and (D) show high correlation coefficients with *R*^2^ > 0.94.

The corresponding DEER measurements of each CW EPR sample exhibit the well-known increase in modulation depths up to values of about Δ ≈ 0.7 (Figure 9A). The resulting distance distributions in Figure 9B also exhibit an overestimation of short distances to quite some extent for *N*_L_ > 2, as it was shown in.^42^ The main characteristics of these 16-DSA-derived distance distributions are now termed as *P*_A_(*r*) for the peak at *r*_A_ = 3.5 ± 0.1 nm and *P*_B_(*r*) for the peak at *r*_B_ = 2.2 ± 0.2 nm that are used for the construction of the relative intensity ratio *P*_AB_(*r*) = *P*_A_(*r*)/P_B_(*r*). As this *P*_AB_(*r*) value may cover a wide range of several orders of magnitude, it is depicted as its natural logarithm in Figure 9C (ln*P*_AB_(*r*)). Generally, the first moment or average distance value ⟨*r*⟩ of the complete distance distribution can be used as an assessment measure for rather broad and ambiguous distance distributions.^52,85^ This first moment ⟨*r*⟩ does not only contain information about the weight center of *P*(*r*), but also reflects slight shifts of *r*_A_ and *r*_B_ for up to about 0.4 nm that might indicate allosteric reorganizations of individual binding pockets upon 16-DSA ligand loading.^5,62,86^ The above-described lock-pin behavior has been reported to increase the albumin diameter by about 0.5 nm through a relative rotation of domains I and II.^62^ Instead, this effect is here observed as a general decrease of FA interspin distances acting like a shoelace that ties the domains together. Hence, ln*P*_AB_(*r*) mainly represents the rise in relative intensity changes of *P*_B_(*r*), depending on the number of equivalent 16-DSA molecules per HSA. A quite smooth curve is obtained for ln*P*_AB_(*r*) that can be fitted with an exponential function (eq S41 in the SI). From these data, an empirical formula can be derived that correlates ln*P*_AB_(*r*) with the FA content *N*_*P*(*r*)_ by
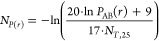
9where ln*P*_AB_(*r*) is the experimental parameter from the distance distribution. A quick and intuitive derivation of eq 9 and fit parameters are shown in the Supporting Information (Table S8). The modulation depth Δ also increases in a nonlinear fashion to a saturation-like state above *N*_Δ_ > 3.5 (Figure 9D). A mathematical model is here adopted for 16-DSA adsorption to HSA (eq 10) in analogy to a Langmuir isotherm that is usually applied to describe gas absorption to energetically heterogeneous surfaces^87^
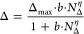
10

Thus, the number of 16-DSA molecules that are absorbed by HSA can be determined by modulation depths from DEER experiments using the following empirical expression
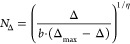
11

Mathematically, the parameters *b* = 0.503 and Δ_max_ = 0.712 (see Table S9) are equivalent to the Langmuir constants, and η = 2.203 is a stretching factor that originally considers energetic inhomogeneities on absorbing surfaces. In our application eq 11 directly correlates modulation depth to the number of occupied ligand binding sites of each HSA molecule.

However, these spin counting strategies are intrinsically restricted and are only applicable in the range of about 0 < *N*_L_ < 4 equiv of 16-DSA due to the saturation-like behavior in the corresponding ln*P*_AB_(*r*) and modulation depth (Δ) curves (Figure 9C,D). Thus, in combination with double integration in CW EPR, eqs 9 and 11 allow for spin counting in an empirical fashion, without accounting for multispin contributions stemming from more than two dipolar-coupled spins in the relevant distance range, which we have studied in HSA before.^42^

**Figure 10:**
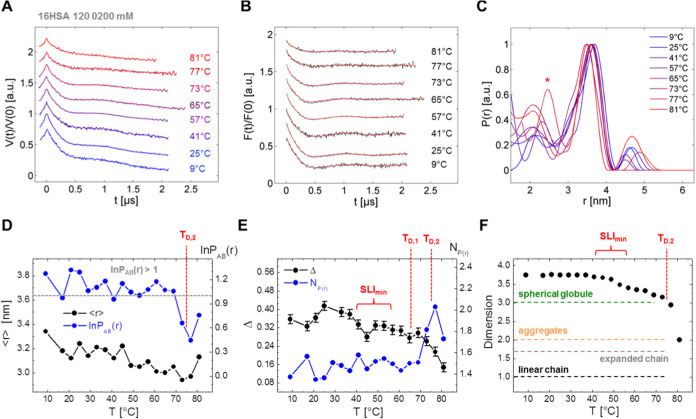
DEER results from temperature denaturation of 16-DSA-probed HSA solutions. (A) Several temperature-dependent raw DEER time traces *V*(*t*)/*V*(0) of 16-DSA-probed HSA (2:1), incubated at selected temperatures with corresponding (B) dipolar evolution functions *F*(*t*)/*F*(0) (black) with regularized fit curves (red) and (C) distance distributions *P*(*r*). The red asterisk in (C) highlights the distribution shape at 77 °C. (D) The first moments ⟨*r*⟩ (black) of *P*(*r*) and ln*P*_AB_(*r*) (blue) are shown as functions of temperature. The gray dotted line indicates ln*P*_AB_(*r*) = 1. (E) Experimental modulation depths Δ and *N*_*P*(*r*)_ (from eq 9) are presented as functions of temperature. Error bars for individual modulation depths were chosen as ΔΔ = 0.02 as suggested in Bode et al.^88^ (F) Temperature-dependent background dimensionality change of 16-DSA-probed HSA. The different typical dimensionality regimes are denoted to indicate shapes of homogeneous and spherical globules (*D* = 3), aggregates/oligomers (*D* = 2), expanded chains (*D* = 1.67) and linear chains.^44,52^ The red items in D–F indicate *T*_D,1_ = 65.5 °C as the first and *T*_D,2_ = 74.9 °C as the second melting temperature from DSC results, calculated from eqs S38 and S39 in the Supporting Information, the corresponding loading ratio is *N*_*P*(*r*)_ = 1.47. SLI_min_ is here given as the temperature range from DLS data in Figure 3D where HSA elongates and the subdomains are assumed to decouple.

To check for the proposed ligand redistribution processes above 64 °C^53^ we chose a rather unconventional strategy whose feasibility will be demonstrated in the following. Aliquots from a single 16-DSA-probed HSA stock solution were incubated for 5 min at a respective temperature between 9 and 81 °C. If cooling proceeds in equilibrium with the environment, all ensembles should be identical and should reflect the ensemble at the glass-transition temperature. We chose shock-freezing from the respective temperature directly—a nonequilibrium process to achieve freezing in a spin ensemble that might be representative of a snapshot of the ensemble at the respective incubation temperature. The results from these experiments are shown in Figure 10. At first sight, the DEER time traces and dipolar evolution functions in Figure 10A,B do not seem to change considerably upon heating. A further interesting aspect is that also the distance distributions do not change decisively (Figure 10C), not even for higher temperatures. However, close inspection of the general distribution shape reveals a slight but traceable increase in *P*_B_(*r*). Thus, the empirical rules of thumb in eqs 9 and 11 are now applied. The resulting values of ln*P*_AB_(*r*) are shown in Figure 10D.

An average value of ln*P*_AB_(*r*) = 1.13 ± 0.12 is obtained as a fingerprint for “native” HSA between ∼9 to 65 °C. Additionally, individual data points are almost entirely located above ln*P*_AB_(*r*) > 1, i.e., when the relative peak ratio is *P*_AB_(*r*) ≈ 3. Above 70 °C, all ln*P*_AB_(*r*) < 1, indicating an increase of the *P*_B_(*r*) feature in the range of 73 to 81 °C, as marked with an asterisk in Figure 10C. The minimum value for ln*P*_AB_(*r*) is found at 77 °C indicating a maximum intensity in *P*_B_(*r*), almost perfectly coinciding with *T*_D,2_ from DSC experiments. In contrast, the first moment ⟨*r*⟩ of *P*(*r*) does not show significant changes apart from a slight decrease in the course of HSA temperature denaturation.

The calculation of individual, temperature-dependent *N*_*P*(*r*)_ values is carried out according to eq 9 and is shown in Figure 10E. An average value of *N*_*P*(*r*)_ = 1.47 ± 0.07 is obtained in the temperature range from 9 to 65 °C. Like for ln*P*_AB_(*r*), the maximum number *N*_*P*(*r*),max_ = 2.03 of 16-DSA ligands per HSA is found at 77 °C. This must now be associated with an increase in FA loading in the still intact HSA molecules, or associated binding sites as caused by ligand redistribution. The fit curves of the DSC-derived melting peaks in Figure 8B deliver corresponding 16-DSA melting peak positions (*T*_D,1_ = 65.5 °C and *T*_D,2_ = 74.9 °C) which are also denoted in Figure 10D–F.

While the *N*_*P*(*r*)_ and ln*P*_AB_(*r*) have features coinciding with *T*_D,2_ quite well, the temperature-dependent modulation depths only show subtle features. An overall maximum modulation depth of Δ = 0.41 is found at 25 °C and at 45 °C a first local minimum appears in the temperature range that coincides with the drop in SLI from DLS experiments (SLI_min_ ≈ 40 – 55 °C) reflecting the loss in protein compactness. A slight kink is also seen for *T*_D,1_. For *T* > 70 °C, Δ *s*ignificantly decreases steadily after passing *T*_D,2_ indicating transient loss toward total loss of HSA compactness or at least an increased contribution of unmodulated background. This can be seen as being caused by the release of ligands, or the ongoing transition from well-defined strongly interacting to more loose and flexible binding states. It would be of interest to analyze whether the same effects occur when the DOXYL group is located deeper inside of the binding tunnel, namely by exchanging the spin probe 16-DSA by 5-DSA.

The background dimensionality of the raw DEER time traces was also adjusted carefully to extract additional qualitative information. A typical value of *D* = 3.74 could be used for HSA at lower incubation temperatures (*T <* 40 °C).^3,41,44^ As this parameter is sensitive to changes in global shape and excluded volume,^44^ successful analyses of available DEER data are only possible when dimensionality is adjusted between 3.0 and 3.7 for temperatures between 40 and 70 °C. Besides the sharp dimensionality drop beyond *T*_D,2_ (*D* < 3), indicating transition to more linearly stretched albumin polypeptide chains,^44,89^ data evaluation is strongly hampered and proper spin echo formation vanishes, likely due to denatured protein in gel-like fractions (see also Figure 2C).^7^ Inhomogeneous vitrification due to protein denaturation from incubations above 81 °C render DEER experiments increasingly inaccessible. Note that the dimensionality curve in Figure 10F resembles an inverted SLI curve (Figure 3D).

## Conclusions

**Scheme 1:**
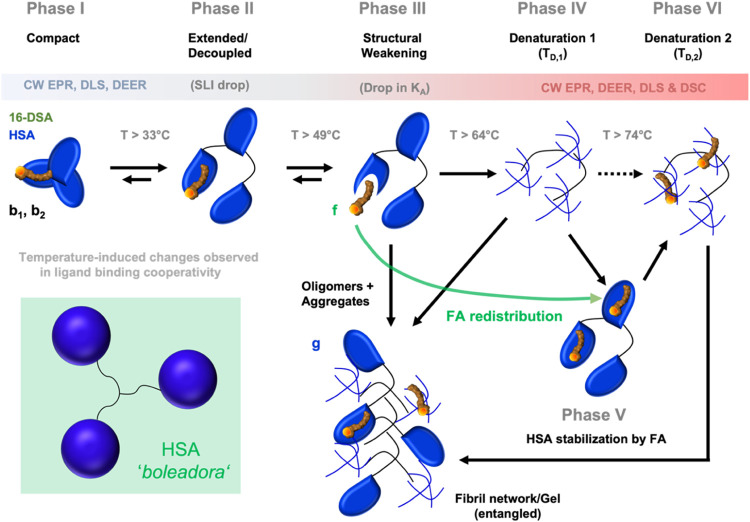
Temperature-Induced Dynamic Phases in HSA Solutions Containing 16-DSA A Model Was Devised from Available Data in this Study that also Corroborates Prevalent Literature. The Temperature-Dependent Behavior can be Separated in Six Phases (I–VI), whereas the First Three Phases Belong to the Proposed Boleadora-Type Dynamics Model of Globular HSA (Phase I). HSA Elongates/Decouples to a Kind of *Boleadora*-like Appearance for *T* > 33 °C (Phase II), Decreases in Structural Integrity that Leads to a Drop in *K*_A_ (*f*) and the Formation of a Fibril Network/Gel Phase for *T* > 49 °C (*g*, Phase III). During the Transition from Phase I to Phase II, Changes in Ligand Binding Cooperativity can be Observed. The Presentation of the Fibril Network is Shown as Proposed by Bhattacharya et al.^96^ The First Denaturation Phase Occurs in Phase IV above 64 °C. The Still Intact HSA Molecule Fraction can be Further Stabilized (Phase V) by Uptake of the FA Ligands that are Released from Denatured HSA Molecules during Phase III and Phase IV. Above 74 °C All HSA Proteins Are Denatured and Accumulate in the Gel Phase (Phase VI).

The observed dynamic processes from 16-DSA-probed HSA solutions in EPR spectroscopy, DLS and DSC are summarized in Scheme 1 (loading ratio 1:1). Overall, six different dynamic rearrangement phases of HSA are encountered in this study. The compact and native state of HSA is clearly observed in the temperature range from about 5 to 40 °C and is termed as Phase I. At *Τ >* 33 °C the protein structure starts to extend as detected by a drop in scattered light intensity (SLI) in DLS experiments (Figure 3D). Simultaneously, the Brownian diffusion component *b*_1_ in corresponding EPR spectra shows a nonlinear decrease in rotational correlation times (Figure 3A), unlike the free diffusion component *b*_2_. Both applied methods, CW EPR and DLS, confirm a protein-based structural extension or decoupling mechanism without any detectable phase transition in DSC. This phenomenon in Phase II can be described as a *rotational decoupling of domains* in HSA, leading to a *boleadora*-type domain arrangement. This temperature-induced domain decoupling was proposed in several other studies,^90,91^ usually referred to as going along with mild alterations in secondary structure.^60,61^ The existence of a ligand-independent opened and closed state of albumin^92^ cannot be confirmed from the obtained data, as the time scale of this process (300 ms) by far exceeds the nanosecond time scale.^93^ However, the energetic contributions from FA binding to HSA (Figure 6) are quite similar to values that were reported in the kinetic model of a two-step attachment profile given by Scheider.^94^

In phase III, the ligand association constant *K*_A_ experiences a detectable drop above 53 °C as the HSA molecule shows increased FA release (*f*), indicative of a structural weakening. At the same time a gel fraction (*g*) appears in CW EPR spectra, formed by aggregation of individual proteins as confirmed by a spontaneous increase in SLI at this temperature. It is noteworthy that Banerjee and Pal^95^ found a structural transition in BSA at 54 °C by differential thermal analysis (DTA) that could be also related to slight changes in DLS data. Altogether, four coexisting dynamic components are observed in CW EPR spectra of 16-DSA (*b*_1_, *b*_2_, *f* and *g*) with the onset of phase III. The rotational decoupling model can be reconciled with the structural elongation/weakening of HSA in a *boleadora*-type picture. We suggest that this is the reason for a mutual entanglement of (sub)domains, as they accumulate and form gel-like, water-depleted regions that are here probed by amphiphilic 16-DSA ligands. Phase IV sets in above 64 °C and denotes the first denaturation process of HSA (*T*_D,1_). While a fraction of HSA is denatured, 16-DSA release is amplified accordingly.

This emerging free fraction of ligand is now assumed to be absorbed by still intact HSA molecules, however, with lower affinity, leading to a structural stabilization (Phase V) of the still functional HSA molecules. Therefore, these proteins experience a shift in denaturation temperatures *T*_D*,i*_ (Figure 8B) as a consequence of the pharmacoperone property^81^ of the FAs (Phase VI). Finally, after oligomerization sets in via self-capturing HSA boleadoras, ultimately a macroscopic gel is formed that consists of a mixture of denatured protein and intact (sub)domains still harboring 16-DSA ligands.

It is also intriguing that the observed phenomena recur in data from diverse methods. Changes in rotational correlation times in CW EPR coincide with SLI effects in DLS data, as well as changes in hydrodynamic radii (*R*_H,*j*_). Additionally, even DEER data exhibit sensitivities to these observed hydrodynamic effects in CW EPR and DLS.

Besides the modulation depth (Δ) that can be viewed as a measure of the overall compactness of the protein ensemble (Figure 10E), the background dimensionality (*D*) qualitatively resembles an inverted shape of the SLI curve from DLS results (Figures 3D and 10F). In particular CW EPR data reveal sophisticated properties in the interconversion thermodynamics that indirectly reflect phase transitions as obtained from DSC, however, on the nanoscopic level of ligand solvation. In this study, it was thus shown that the strategies that were developed for the calculation of interconversion processes (*K*_IC_) in core–shell polymers can also contribute to a better nanothermodynamic understanding of much more complex systems such as HSA.

Ligand hydration states can clearly be obtained from Δ*C*_P,IC_. As it was predicted earlier, such hydration interactions exhibit complicated temperature dependences of Δ*C*_P_.^97^ This is in line with the findings made here (Figure 7). The obtained apolar dehydration temperatures *T*_AD,1_ = 53.6 °C and *T*_AD,2_ = 82.7 °C denote the onset and the termination of the denaturation process in HSA. Furthermore, the region around the apolar hydration temperature *T*_AH,1_ = 69.4 °C most probably describes the thermodynamically stabilizing process by ligand reorganization in the system that ultimately leads to the appearance of a second denaturation temperature *T*_D,2_ (Figure 8). The derived thermodynamic quantities show how intricately ligands and protein are energetically coupled. In particular, the heat capacity curves of HSA (Δ*C*°_P,HSA_, from DSC) and 16-DSA interconversion (Δ*C*°_P,IC_, from CW EPR), respectively, are almost directly complementary.

The predicted second denaturation temperature from DSC experiments (*T*_D,2_ = 74.4 °C) coincides quite well with the value obtained from the van’t Hoff plot of ln* K*_A_ (*T*_m_ = 75.3 ± 0.9 °C) for identical loading ratios (1.13 equiv of 16-DSA). With increasing 16-DSA:HSA ratio, ln* K*_IC_ from CW EPR data (Figure 8C), Δ *f*rom DEER data (Figure 9D), and *T*_D,2_ from DSC experiments (Figure 8B) all reach a plateau value, indicating that structural stabilization experiences a saturation at about 3.5 < *N*_L_ < 5.0 bound FA equivalents. An ITC study of Fang et al.^98^ revealed that only 5 binding sites contribute to structural changes that accompany FA binding (in this case: myristate). From all this, one may conclude that these plateau-like regions above *N*_L_ > 3.5 illustrate allosteric reorganizations of HSA reducing its flexibility. Experimental parameters ln* K*_IC_, Δ, and also SLI values can be seen to truly probe for protein compactness and flexibility. The bound (paramagnetic) FAs can be understood as effective intrinsic melting temperature-increasing equivalents (*T*_D,1,min_ + 1.28 °C·*N*_L_). These FAs contribute to HSA’s stability solely upon binding and are therefore considered as physiological pharmacoperones.

DEER data gave experimental evidence for FA ligand redistribution at higher temperatures. This effect leads to a slight, yet detectable change in distance distribution characteristics (ln*P*_AB_(*r*)) and is indicative of higher ligand binding site occupation per natively folded albumin (ca. +40%).

Our data and the discussion in light of previous knowledge suggest that the established but simple picture of weak and strong binding sites in HSA has to be extended and revised. We suggest that protein compactness strongly affects the mode of diffusion of bound ligand. It has to be further ascertained to which extent ligand binding cooperativity is affected by the rotational decoupling of HSA subdomains. From Figure 4A and S5A it appears as if not only temperature, but also the associated domain proximity has an effect on the nature of ligand binding cooperativity. A similar temperature effect was also observed in laurate and myristate binding to HSA by Pedersen et al.^99^ The FA-lock pin hypothesis given by Curry^62^ can be confirmed by ln* K*_IC_ at different 16-DSA loadings and temperatures (Figure 8C).

In summary, it should be emphasized that the stability of HSA is not only induced by the sheer presence of FA ligands, but is also energetically driven from the interconvertible modes of intrinsic FA diffusion that facilitate storage of thermal energy in form of rotational entropy. As we have described, e.g., in,^7^ gel formation in HSA and BSA can take place below the denaturation temperature. The boleadora-type rotational model visualizes the partial unfolding into three rotationally decoupled domains that then interact and finally lead to protein entanglement and gel formation.

In future studies, we will transfer this EPR-spectroscopic approach to other ligand binding proteins and especially test whether the pharmacoperone-activity of bound ligands is observable and potentially functionally important there, too. This will include proteins of the FA binding protein (FABP) family, for which we have observed interconversion of prebinding and strong binding of FAs.^100^

## Supporting Information Available

Further Sample Preparation Details, Determination of the Critical Micelle Concentration (CMC) of 16-DSA, CW EPR Simulations and Parameters, Scatchard Plot Construction, Fit Parameters of ln* K*_IC_ of 16-DSA bound to HSA, Derivation of Thermodynamic Functions of ln* K*_IC_, Fitting Function from Temperature Stability Shifts in DSC and ln* K*_IC_, Spin Counting and Fitting Functions from DEER-derived Parameters ln*P*_AB_(*r*) and Δ *a*s Functions of 16-DSA Loading (PDF)
