# Supplementary material for: Human Serum Albumin Loaded with Fatty Acids Reveals Complex Protein–Ligand Thermodynamics and Boleadora-Type Solution Dynamics Leading to Gelation
Source: J Phys Chem B. 2025 Mar 26;129(14):3571–89. doi: 10.1021/acs.jpcb.4c08717 (PMC11995378; doi:10.1021/acs.jpcb.4c08717)
Supplement: Supplementary file 1 — jp4c08717_si_001.pdf [file jp4c08717_si_001.pdf]

Human Serum Albumin Loaded with Fatty Acids Reveals Complex Protein-Ligand Thermodynamics and Boleadora-Type Solution Dynamics Leading to Gelation

Jörg Reichenwallner,[€,#] **Sebastian Michler,** [€] **Christian Schwieger,** [€] **Dariush Hinderberger**[€]*

[€]Institute of Chemistry, Physical Chemistry – Complex Self-Organizing Systems, Martin Luther University Halle-Wittenberg, Von-Danckelmann-Platz 4, 06120 Halle (Saale), Germany

# present address: Department of Biochemistry, University of Toronto, Toronto, ON, M5S 1A8, Canada.

* to whom correspondence should be addressed, dariush.hinderberger@chemie.uni-halle.de

Supporting Information

Contents

S1 | Preparation of 0.137 M DPBS Solution at pH 7.4 S2

S2 | Critical Micelle Concentration (CMC) of 16-DSA in DPBS pH 7.4 at $T = 25°C$ S2

S3 | EPR simulation parameters from 16-DSA binding to HSA S5

S4 | Spectral Simulations of CW EPR Data for Scatchard Plot Construction S7

S5 | Assessing Appropriate Fit Parameters from $\ln K_{IC,x}$ of 16-DSA Bound to HSA S10

S6 | Derivation of Thermodynamic Functions from $\ln K_{IC,j}$ in equation 3 S11

S7 | Fit Curves from Temperature Stability Shifts in DSC and $\ln K_{IC,j}$ S16

S8 | Spin Counting and experimental DEER data for deriving Parameters $\ln P_{AB}(r)$ and Δ as Functions of 16-DSA Loading. S17

S9 | Supporting References S19

S1 | Preparation of 0.137 M DPBS Solution at pH 7.4

The accomplishment of producing 1000 ml DPBS buffer[S1] at pH 7.4 as an essential isotonic ingredient for all samples is given in the following. Initially, three different solutions are prepared referred to as solution A – C. Preparation of solution A comprises dissolving 8.00 g of sodium chloride (NaCl, Sigma-Aldrich), 0.20 g potassium chloride (KCl, Merck), 1.15 g disodium phosphate (Na_2HPO_4, Sigma-Aldrich) and 0.20 g potassium dihydrogenphosphate (KH_2PO_4, Fluka) in 700 ml ultrapure water (Milli-Q). Solution B contains 0.10 g calcium chloride ($CaCl_2$, Carl Roth) and solution C contains 0.10 g magnesium chloride hexahydrate ($MgCl_2.6H_20$, Santa Cruz Biotechnology). Both solutions B and C are then separately prepared by dissolving each salt in 100 ml of ultrapure water (Milli-Q). All three solutions were filtered over 0.45 µm Millex-LCR PTFE membranes (Merck Millipore®) before they were autoclaved separately to prevent precipitation of these salts. After cooling, solutions A – C are mixed (900 ml) and titrated to pH 7.4 with 1 M hydrochloric acid (HCl, Fisher Scientific) or 1 M sodium hydroxide (NaOH, Fisher Scientific), while the solution is stirred with gentle agitation. Again, about 100 ml of ultrapure water is added and a final pH measurement is taken. The ready-made solution is stored at 4 °C and may be used for up to 12 months without any concern. The solution may be regarded as expired, when cloudy aggregates have formed. The final osmolality (310.6 mosmol/l) is considered as the sum concentration of all osmotically active ion particles in the solution, i.e. 136.89 mM NaCl, 2.68 mM KCl, 8.1 mM Na_2HPO_4, 1.47 mM KH_2PO_4, 0.90 mM $CaCl_2$ and 0.49 mM $MgCl_2 \cdot 6H_2O$.

S2 | Critical Micelle Concentration (CMC) of 16-DSA in DPBS pH 7.4 at *T* = 25 °C

It has been shown in earlier publications that obtaining CMC values for 16-DSA is challenging due to the highly complex solution behavior of this molecule. Generally, the 16-DSA spin probe is added to buffer solutions to detect CMCs and polarity effects of host molecules[S2-S4] by determination of the rotational correlation time τ_c. This value is most sensitive to changes in microviscosity, which can change drastically upon structural rearrangements in solution at reasonable host concentrations. Here, microviscosity is not altered considerably when 16-DSA concentration is increased and micelle formation is triggered (τ_c = 68 ps for 32 µM 16-DSA to τ_c = 99 ps for 2980 µM 16-DSA), so an alternative way of interpreting data has to be figured out.

As 5-DSA was reported to have a CMC of 0.035 mM in saline phosphate buffer at 37 °C,[S5] 16-DSA can be expected to have a very similar value as it also contains stearic acid as an identical molecular matrix. Here, the commonly accepted inherent spectroscopic problems associated with spin-labeled DSA molecules are surmounted by insistent sample preparation until an adequate set of measurements is obtained. CW EPR spectra were obtained here in the concentration range from 13 – 3098 µM without a reasonable gap between individual data points that would give rise to a lack of information about the onset of micelle formation. Unlike in Rehfeld *et al.*[S5] an alternative semi-empirical method of detecting the abundance of micelles in our samples is employed. As the exchange-broadened micellar spectral component grows with increasing 16-DSA concentration,[S6] the relative peak intensities of the center-

field peak of freely tumbling 16-DSA (h_0) and micellar spectral components of 16-DSA (h_M) are compared. As all experimental parameters are kept constant in the sample (T, pH, ionic strength, solvent), the reproducibility of individual measurements is still overwhelmingly restricted. Therefore, double integration of individual CW EPR spectra was used as a quantitative spin counting tool. While the micellar spectral component h_M may partially overlap with the ^{13}C-satellite signals at the central ^{14}N-nitroxide line ($m_I = 0$), the ^{13}C intensity (h_C) is subtracted by default to obtain $h_{M,0} = h_M - h_C$ that allows to build the formal ratio $h_{M,0}/h_0$ (**Figure S1**). Thereupon, this value is plotted versus the 16-DSA concentration as extracted from double integration.

As it is not always possible to prepare all the samples from the same stock solution it is strongly recommended to double integrate all stock solution CW EPR spectra additionally and normalize them against each other. The reason is that obviously not all supplier lots contain similar amounts of spin-bearing 16-DSA molecules. In this case an exemplary procedure would be to calculate:

$$r_{DI} = \frac{\iint S_{\text{stock1}}^{\text{16-DSA}}(B)\mathrm{d}^2B}{\iint S_{\text{stock2}}^{\text{16-DSA}}(B)\mathrm{d}^2B} = 0.69 \qquad \text{(S1).}$$

This routine applied well here to align independent measurements from two different stock solutions by an appropriate signal strength normalization factor r_{DI}.

It has been shown that a concise kink is emerging in the curve at the onset of micelle formation, as it is generally encountered in e.g. surface tension measurements.[S7] Linear fits of data points lying on straight lines following the relation $c_i = c_{k,i} \cdot x + c_{0,i}$ enable to extrapolate an intercept point of both lines that directly yields the CMC value for 16-DSA. In this case it can be shown that the relation:

$$\text{CMC} = c_{k,1} \cdot \left(\frac{c_{0,2} - c_{0,1}}{c_{k,1} - c_{k,2}} \right) + c_{0,1} \qquad \text{(S2)}$$

holds, if $i = 1$ corresponds to the low concentration line ($c_{\text{16-DSA}}$ < CMC) and $i = 2$ corresponds to the high concentration line ($c_{\text{16-DSA}}$ > CMC), whereas $c_{k,i}$ and $c_{0,i}$ are the individual slopes and y-axis intercepts, respectively.

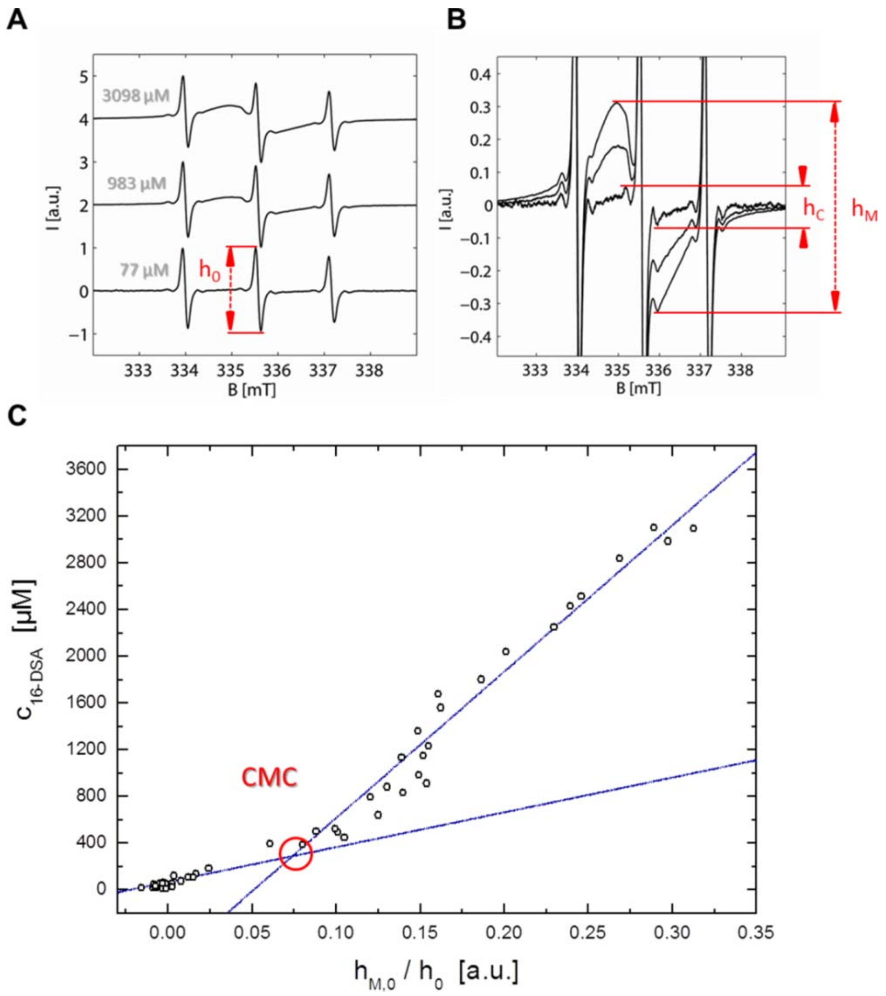

Figure S1 | Graphical determination of the CMC of 16-DSA from CW EPR experiments. Micelle formation propensity gets ever stronger with increasing 16-DSA concentration. Therefore, some exemplary CW EPR spectra are shown for 16-DSA concentrations at (**A**) 77 µM, 983 µM and 3098 µM. Here, the center-field line height (h_0) is highlighted in red. (**B**) Due to micelle formation broad spectral characteristics appear that can be characterized with the micelle feature (h_M). Additionally, the ^{13}C signal height (h_C) is subtracted from h_M, so that $h_{M,0} = h_M - h_C$. (**C**) Plot of $c_{16\text{-DSA}}$ versus the ratio $h_{M,0}/h_M$. The intersection region of both lines is defined as the critical micelle concentration (CMC, red circle).

The formation of 16-DSA micelles in 0.137 M DPBS buffer at pH 7.4 and T = 25 °C as determined from **Figure S1C** is therefore CMC = (285 ± 29) µM. Most of the subsequent samples were therefore supplied with about 0.2 mM 16-DSA to prevent micelle formation and unnecessary complication of spectral analysis, while a still sufficiently strong signal can be obtained from the sample. However, the determination of an exchange constant k_e leading to a line broadening as described in Molin *et al.*[S6] is not accessible by this method. Upon keeping the DPBS buffer concentration constant the influence of electrolyte concentration on the CMC value is largely excluded.[S8]

S3 | CW EPR simulation parameters from 16-DSA binding to HSA

A detailed strategy about how to simulate temperature-dependent multicomponent spectra from 16-DSA has been thoroughly explained in Reichenwallner et al. [S9] The general approach for identifying sub-spectra in the decomposition of recorded CW EPR spectra of 16-DSA-probed HSA solutions is summarized in **Figure S2**. Exemplary simulation parameters are given in **Table S1** and the simulation of CW EPR spectra for all temperatures from 5 – 97 °C in steps of 4 °C are presented in **Figure S3**.

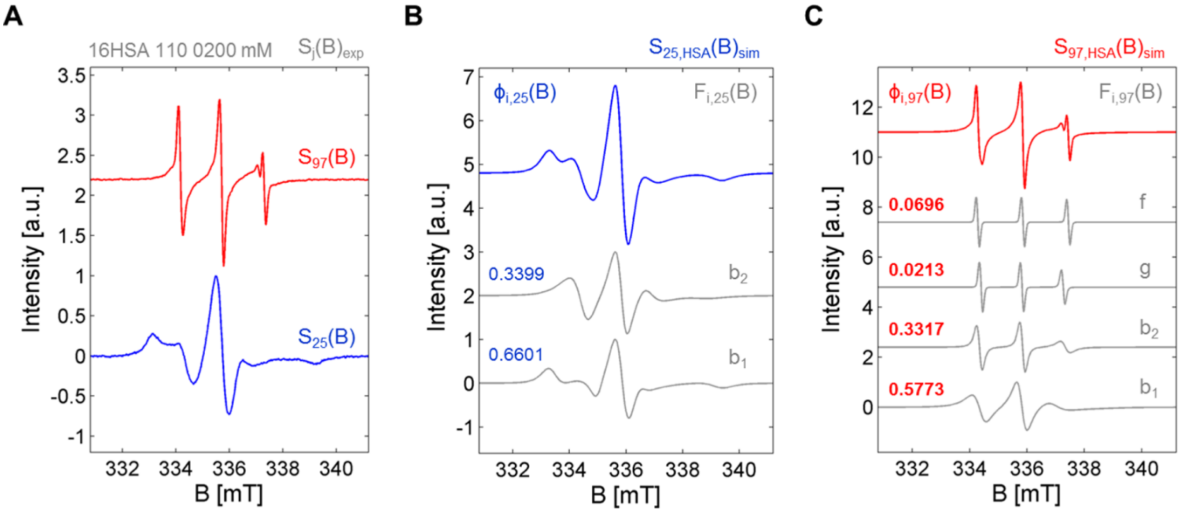

Figure S2 | Decomposition strategy of CW EPR spectra from 16-DSA-probed HSA solutions. (**A**) The temperature-dependent experimental CW EPR spectra *S(B)* are shown for 25 °C (blue) and 97 °C (red). (**B**) The spectral simulations of the CW EPR spectrum at 25 °C in A) are shown. The fully simulated spectrum (blue) is composed of two sub-spectra b_1 and b_2 that have relative contributions of 66 % (b_1) and 34 % (b_2). (**C**) Spectral simulations of the CW EPR spectrum at 97 °C in A). The full spectrum is composed of four components b_1, b_2, g and f that have relative contributions of 58 % (b_1), 33 % (b_2), 2 % (g) and 7 % (f). All simulation traces of sub-spectra are shown in gray color. All measurements were conducted in 0.137 M DPBS pH 7.4 at an equivalent concentration of $c_{16\text{-DSA}}$ = 0.204 mM (with c_{HSA} = 0.180 mM).

Table S1 | Example simulation parameters of 16-DSA-probed HSA solutions as shown in Fig S2

Sample	T [°C]	*species*	$\phi_{i,j}$ [%]	g_{iso}	a_{iso} [MHz]	a_{iso} [G]	τ_c [ns]	β [°]
**HSA**	25	b_1	66.009	2.00593	42.93	15.25	11.262	16
		b_2	33.991	2.00593	42.93	15.25	4.319	45
	37	b_1	56.302	2.00593	42.87	15.23	9.982	16
		b_2	43.698	2.00593	42.87	15.23	3.808	45
	97	b_1	57.734	2.00593	42.33	15.04	2.928	16
		b_2	33.172	2.00593	42.33	15.04	1.119	45
		f	6.960	2.00587	44.33	15.75	0.021	45
		g	2.133	2.00607	40.20	14.28	0.137	45

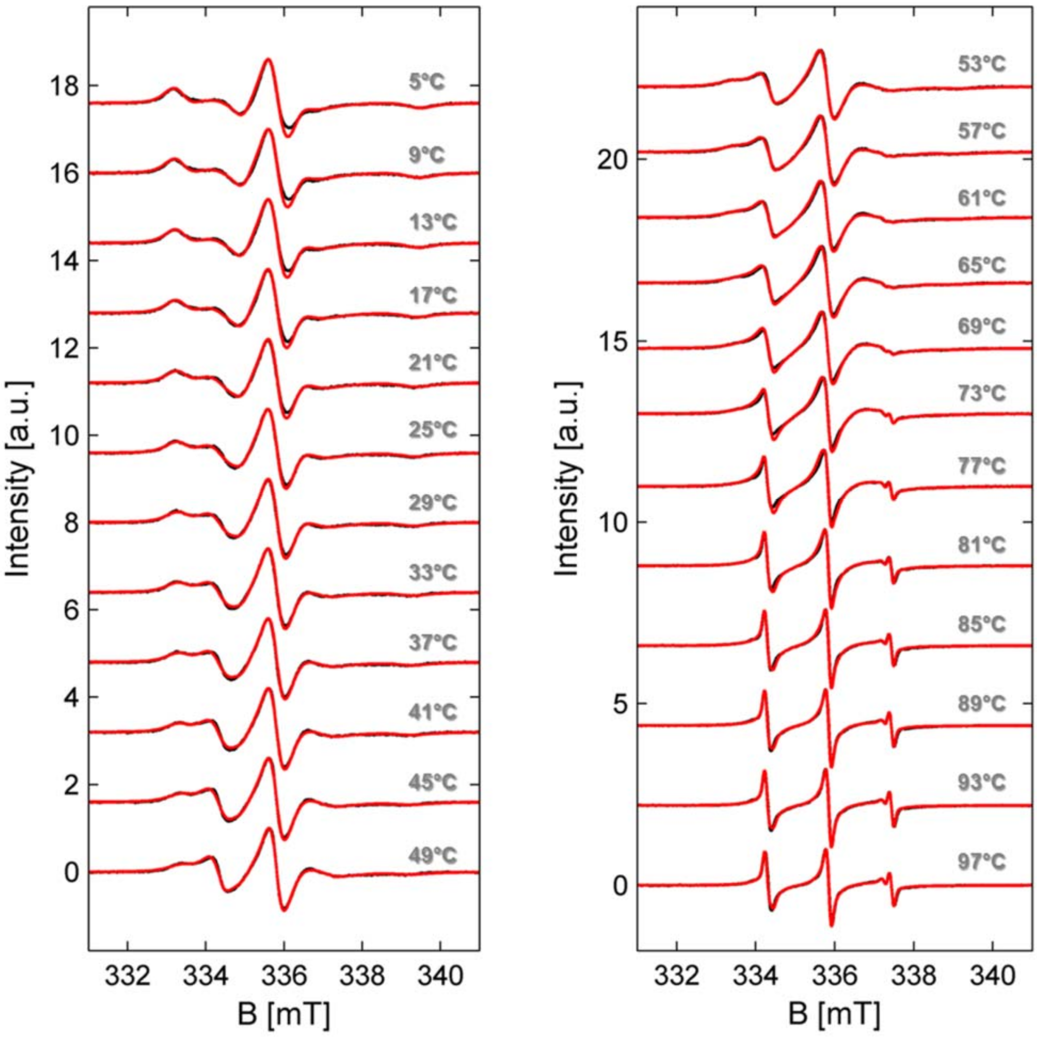

Figure S3 | Temperature-dependent spectral simulations of 16-DSA-probed HSA solutions. All CW EPR simulations (for simulation parameters see Table S1) of 16-DSA-probed HSA solutions are shown in the temperature range from 5°C - 97°C. Experimental data are shown in black and simulations in red.

S4 | Spectral Simulations of CW EPR Data for Scatchard Plot Construction

All spectral simulations for temperature-dependent Scatchard plots[S10] of 16-DSA-probed HSA solutions are shown in **Figure S4**. A starting set of simulation parameters is given in **Table S1**.

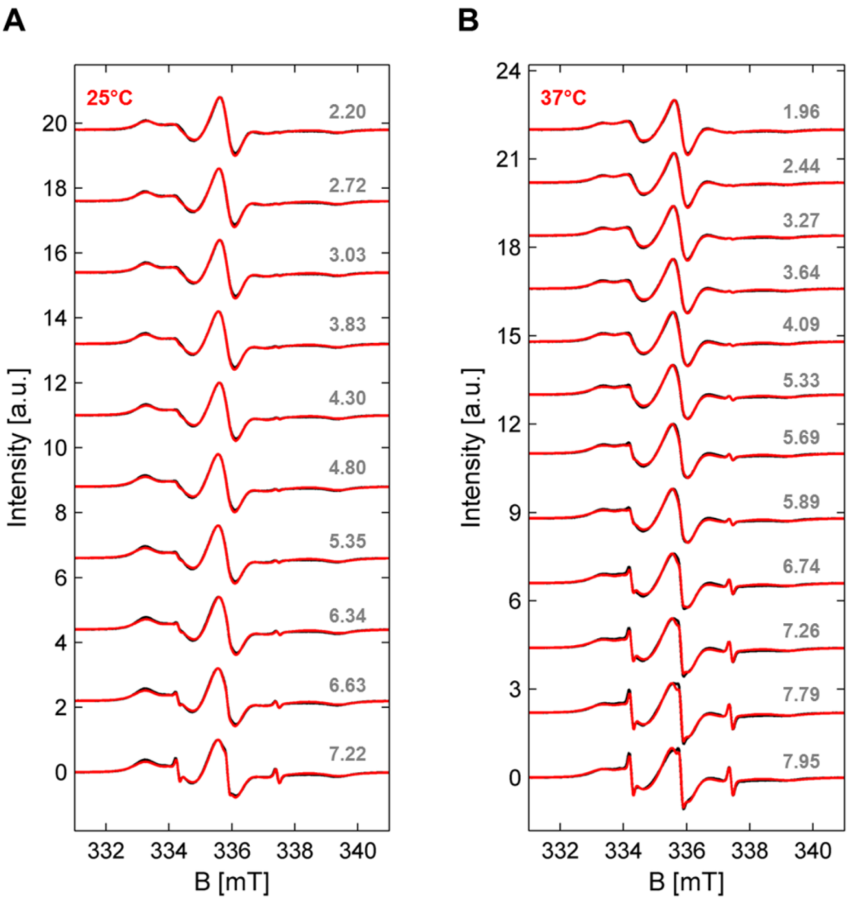

Figure S4 | Concentration-dependent spectral simulations of 16-DSA-probed HSA solutions. All CW EPR spectral simulations of 16-DSA-probed HSA solutions are shown in the loading range from about 2:1 to 8:1 at (**A**) 25°C and (**B**) 37°C. Experimental data are shown in black and simulations in red.

The Scatchard evaluation in **Figure S5A** was conducted according to equations S3-S5. Parameters from the corresponding linear fit are shown in **Table S2**. Generally, [L] is denoted as the ligand concentration. Subscript indices of [L] can be understood as giving specific concentrations in the b = bound and f = free state. Index t refers to the total ligand concentration.

$$\frac{[\mathrm{L}]_{b,k}}{[\mathrm{L}]_{f,k}} = \frac{[\mathrm{L}]_{t,k} - [\mathrm{L}]_{f,k}}{[\mathrm{L}]_{f,k}} = -\frac{1}{K_{\mathrm{D},k}} \cdot \left([\mathrm{L}]_{b,k} - [\mathrm{L}]_{b,k,y=0} \right) \tag{S3}$$

$$\nu = -\frac{1}{K_{D,k}}\left(N_L - N_{E,k}\right) = -K_{A,k}\left(N_L - N_{E,k}\right) \tag{S4}$$

$$y = \nu = b \cdot x + a = -K_A \cdot (N_L - N_E) + \nu_{N_L \to 0} \tag{S5}$$

Table S2 | Fit parameters from linear Scatchard plot phases of 16-DSA interacting with HSA

Parameter	K_A [M^{-1}]	$\nu_{NL\to 0} = N_E \cdot K_A$ [µM^{-1}]	q^a	R^2
$j = 25$	$(1.65889 \pm 0.04274) \cdot 10^6$	13.46333 ± 0.15949	1–10	0.99406
$j = 37$	$(3.5565 \pm 0.6295) \cdot 10^5$	3.08908 ± 0.45300	9–11	0.93925

$^a q$ = data point range in **Figure S5A** where the fit curve was adjusted to.

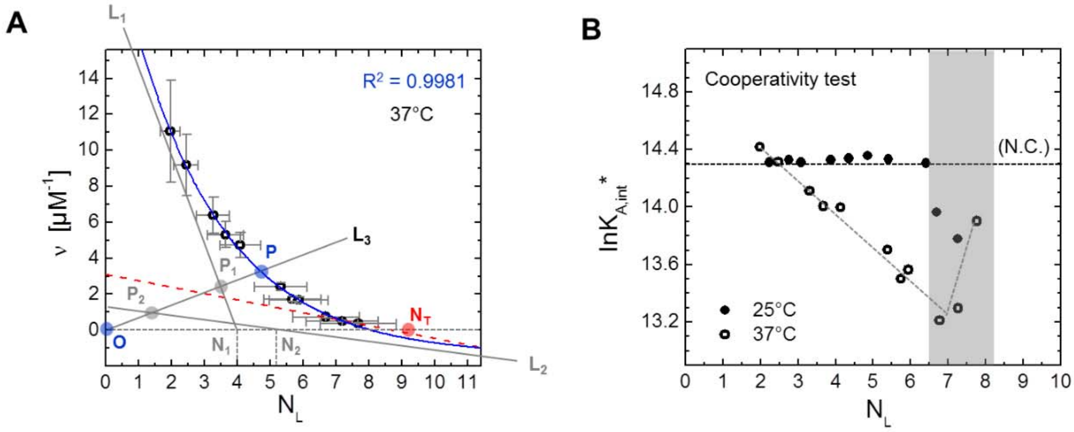

Figure S5 | 16-DSA binding affinity and cooperativity of HSA. Scatchard plot of 16-DSA interacting with HSA at (**A**) 37 °C (black, open circles) with an arbitrary exponential fit curve for highlighting the exponential curve progression (blue). The Rosenthal method[S11] was applied where the three lines (L_1, L_2, L_3) allow for the consideration of two groups of binding sites (N_1, N_2). A linear fit (red dotted line) to the last three data points typically gives the total number of binding sites (N_T). The Scatchard plot was constructed in the loading ratios from 2:1 to 8:1. (**B**) A cooperativity test for both Scatchard plots (Fig.4A and Fig.S5A) was performed according to the scheme presented in Reichenwallner et al.[S12] and Henning et al.[S13] The region where the type of cooperativity changes in both curves is highlighted in grey. The change from negative to positive cooperativity at 37 °C (open circles) is overlaid with gray dotted lines as a guide to the eye.

A Scatchard evaluation of **Figure S5B** was not conducted as no linearity was observed in the whole data range. Due to the exponential shape an empirical fit curve was used in order to reproduce the general shape according to the relation:

$$\nu = \nu_{N_L \to 0} \cdot e^{-N_L / B_\nu} + \nu_{N_L \to \infty} \tag{S6}$$

Parameters from this exponential fit curve are shown in **Table S3** and the biphasic Scatchard plot in **Figure S5A** was evaluated according to the Rosenthal method.[S11] The results from this approach are summarized in **Table 1**.

Table S3 | Fit parameters from Scatchard plot of 16-DSA interacting with HSA at 37°C

**Parameter**	$\nu_{NL\to 0}$ **[µM⁻¹]**	$\nu_{NL\to\infty}$ **[µM⁻¹]**	B_ν	q^a	R^2
$j = 37$	25.19168 ± 0.67608	–1.42223 ± 0.29311	2.79927 ± 0.15579	1–11	0.9981

[a]q = data point range in **Figure S5A** where the fit curve was adjusted to.

The van't Hoff plot of $\ln K_{A,j}$ in **Figure 4B** was fitted with a Boltzmann-type sigmoidal function as shown in equation 1 (main manuscript). The parameters from this fit are given in **Table S4**.

Table S4 | Fit parameters from $\ln K_{A,j}$ of 16-DSA interacting with HSA from equation 1

**Parameter**	**Value**	**Physical implication**
α_1	8.80431 ± 0.17671	lower bound $\ln K_{A,j}$
α_2	12.89806 ± 0.17763	upper bound $\ln K_{A,j}$
α_3 [K⁻¹]	$(2.87000000 \pm 0.00737929)\cdot 10^{-3}$	midpoint temperature T_m
α_4 [K⁻¹]	$(5.34239 \pm 0.820633)\cdot 10^{-5}$	width of transition

S5 | Assessing Appropriate Fit Parameters from ln$K_{IC,x}$ of 16-DSA Bound to HSA

A non-linear curve fit procedure was applied to the van't Hoff plot of ln$K_{IC,x}$ of 16-DSA bound to HSA as shown in **Figure 5**. Best fits were obtained for a curve shape composed from an exponential and a double Boltzmann function. According fit parameters were obtained with a non-standard home-written origin code:

$$y = y_0 + B \cdot \exp(x / t_1) + A(\text{frac}(1 + \exp((x - x_{0,1}) / k_1)) + (1 - \text{frac})/(1 + \exp((x - x_{0,2}) / k_2))) \quad \text{(S7)}$$

The resulting fit parameters from equation S7 were renamed for equation 3 and the corresponding values are listed in **Table S5**. Due to the high number of fit parameters no error margins could be determined.

Table S5 | Fit parameters from ln$K_{IC,x}$ in equation 3 of 16-DSA bound to HSA

Parameter	Fit parameter	Value	SI unit	Physical implication
ln$K_{IC,0}$	y_0	1.16783	–	y-intercept
κ_1	B	–0.0019	–	–
κ_2	$1/t_1$	2049.524715	K	–
κ_3	A	–1.23637	–	total step width
κ_4	frac	0.54918	–	partial step width
κ_5	$x_{0,1}$	0.00306	K^{-1}	midpoint temperature $T_{m,1} = T_{AD,1}$
κ_6	k_1	$2.80139 \cdot 10^{-5}$	K^{-1}	width of transition 1
κ_7	$x_{0,2}$	0.00281	K^{-1}	midpoint temperature $T_{m,2} = T_{AD,2}$
κ_8	k_2	$1.58927 \cdot 10^{-5}$	K^{-1}	width of transition 2

S6 | Derivation of Thermodynamic Functions from ln$K_{\mathrm{IC},x}$ in equation 3

In principle, the derivation of equations 4, 5 and 7 is straightforward. The main calculations are restricted to the following mathematical rules, as the quotient rule:

$$\frac{\mathrm{d}}{\mathrm{d}x}\left[\frac{u(x)}{v(x)}\right]=\frac{u'(x)\cdot v(x)-u(x)\cdot v'(x)}{v(x)^2} \quad , \tag{S8}$$

the reciprocal rule:

$$\frac{\mathrm{d}}{\mathrm{d}x}\left[\frac{1}{u(x)}\right]=-\frac{u'(x)}{u(x)^2} \quad , \tag{S9}$$

the chain rule:

$$\frac{\mathrm{d}}{\mathrm{d}x}\left[e^{u(x)}\right]=e^{u(x)}\cdot u'(x) \tag{S10}$$

and the power rule:

$$\frac{\mathrm{d}}{\mathrm{d}x}\left[u(x)^n\right]=n\cdot u(x)^{n-1}\cdot u'(x) \quad . \tag{S11}$$

According to equation 4 the calculation of $\Delta H^{\circ}_{\mathrm{IC},x}$ proceeds in the following way:

$$\Delta H^{\circ}_{\mathrm{IC},x}=-R\cdot\frac{\mathrm{d}}{\mathrm{d}x}\left[\ln K_{\mathrm{IC},x}\right] \quad , \tag{S12}$$

whereas

$$\ln K_{\mathrm{IC},x}=\ln K_{\mathrm{IC},0}+\kappa_1\cdot e^{\kappa_2 x}+\frac{\kappa_3\kappa_4}{1+e^{(x-\kappa_5)/\kappa_6}}+\frac{\kappa_3(1-\kappa_4)}{1+e^{(x-\kappa_7)/\kappa_8}} \quad , \tag{S13}$$

as it is shown alternatively in equation 3. Differentiation of ln$K_{\mathrm{IC},x}$ with respect to x leads to:

$$\frac{\mathrm{d}}{\mathrm{d}x}\left[\ln K_{\mathrm{IC},x}\right]=\underbrace{\frac{\mathrm{d}}{\mathrm{d}x}\left[\ln K_{\mathrm{IC},0}\right]}_{0}+\underbrace{\frac{\mathrm{d}}{\mathrm{d}x}\left[\kappa_1\cdot e^{\kappa_2 x}\right]}_{\text{scheme 1}}+\underbrace{\frac{\mathrm{d}}{\mathrm{d}x}\left[\frac{\kappa_3\kappa_4}{1+e^{(x-\kappa_5)/\kappa_6}}\right]}_{\text{scheme 2}}+\underbrace{\frac{\mathrm{d}}{\mathrm{d}x}\left[\frac{\kappa_3(1-\kappa_4)}{1+e^{(x-\kappa_7)/\kappa_8}}\right]}_{\text{scheme 2}} \quad , \tag{S14}$$

where the first term is zero and the second term can be calculated according to scheme 1 in equation S14 that utilizes the chain rule for exponential functions (eq. S10):

$$\frac{\mathrm{d}}{\mathrm{d}x}\left[\kappa_1\cdot e^{\kappa_2 x}\right]=\kappa_1\cdot\frac{\mathrm{d}}{\mathrm{d}x}\left[e^{\kappa_2 x}\right]=\kappa_1\left[\kappa_2 e^{\kappa_2 x}\right]=\kappa_1\kappa_2 e^{\kappa_2 x} \quad . \tag{S15}$$

The third term is calculated according to scheme 2 in equation S14 using the reciprocal rule (eq. S9):

$$\frac{\mathrm{d}}{\mathrm{d}x}\left[\frac{\kappa_3\kappa_4}{1+e^{(x-\kappa_5)/\kappa_6}}\right]=\kappa_3\kappa_4\frac{\mathrm{d}}{\mathrm{d}x}\left[\frac{1}{1+e^{(x-\kappa_5)/\kappa_6}}\right]=\kappa_3\kappa_4\frac{\mathrm{d}}{\mathrm{d}x}\left[\frac{1}{u_1(x)}\right] \quad , \tag{S16}$$

where $$u_1(x)=1+e^{(x-\kappa_5)/\kappa_6} , \tag{S17}$$

$$u_1'(x)=\frac{\mathrm{d}}{\mathrm{d}x}\left[1\right]+\frac{\mathrm{d}}{\mathrm{d}x}\left[e^{(x-\kappa_5)/\kappa_6}\right] \quad , \tag{S18}$$

and $$u_2(x)=\frac{x-\kappa_5}{\kappa_6} \quad \text{and} \quad u_2'(x)=\frac{1}{\kappa_6}\left(\frac{\mathrm{d}}{\mathrm{d}x}\left[x\right]+\frac{\mathrm{d}}{\mathrm{d}x}\left[-\kappa_5\right]\right)=\frac{1}{\kappa_6} . \tag{S19}$$

This leads to: $$u_1'(x)=\frac{\mathrm{d}}{\mathrm{d}x}\left[e^{(x-\kappa_5)/\kappa_6}\right]=\frac{e^{(x-\kappa_5)/\kappa_6}}{\kappa_6} \tag{S20a}$$

and finally it turns out that:

$$\begin{aligned}\frac{\mathrm{d}}{\mathrm{d}x}\left[\frac{\kappa_3\kappa_4}{1+e^{(x-\kappa_5)/\kappa_6}}\right]&=\kappa_3\kappa_4\frac{\mathrm{d}}{\mathrm{d}x}\left[\frac{1}{u_1(x)}\right]=\kappa_3\kappa_4\left[-\frac{u_1'(x)}{u_1(x)^2}\right]=-\kappa_3\kappa_4\left[-\frac{\frac{1}{\kappa_6}(e^{(x-\kappa_5)/\kappa_6})}{(1+e^{(x-\kappa_5)/\kappa_6})^2}\right]\\&=-\frac{\kappa_3\kappa_4}{\kappa_6}\cdot\frac{e^{(x-\kappa_5)/\kappa_6}}{(1+e^{(x-\kappa_5)/\kappa_6})^2}\end{aligned} \quad . \tag{S21a}$$

The same procedure applies to the fourth term with the result:

$$\begin{aligned}\frac{\mathrm{d}}{\mathrm{d}x}\left[\frac{\kappa_3(1-\kappa_4)}{1+e^{(x-\kappa_7)/\kappa_8}}\right]&=\kappa_3(1-\kappa_4)\frac{\mathrm{d}}{\mathrm{d}x}\left[\frac{1}{u_1(x)}\right]=\kappa_3(1-\kappa_4)\left[-\frac{u_1'(x)}{u_1(x)^2}\right]=\kappa_3(1-\kappa_4)\left[-\frac{\frac{1}{\kappa_8}(e^{(x-\kappa_7)/\kappa_8})}{(1+e^{(x-\kappa_7)/\kappa_8})^2}\right]\\&=-\frac{\kappa_3(1-\kappa_4)}{\kappa_8}\cdot\frac{e^{(x-\kappa_7)/\kappa_8}}{(1+e^{(x-\kappa_7)/\kappa_8})^2}\end{aligned} \quad . \tag{S21b}$$

Finally, the expression in equation 5 is obtained:

$$\Delta H^{\circ}_{\mathrm{IC},x}=-R\left(\kappa_1\kappa_2\cdot e^{\kappa_2 x}-\frac{\kappa_3\kappa_4}{\kappa_6}\cdot\frac{e^{(x-\kappa_5)/\kappa_6}}{(1+e^{(x-\kappa_5)/\kappa_6})^2}-\frac{\kappa_3(1-\kappa_4)}{\kappa_8}\cdot\frac{e^{(x-\kappa_7)/\kappa_8}}{(1+e^{(x-\kappa_7)/\kappa_8})^2}\right) . \tag{S22}$$

The free energy of interconversion is given by:

$$\Delta G^{\circ}_{\mathrm{IC},j}=\Delta H^{\circ}_{\mathrm{IC},j}-T\Delta S^{\circ}_{\mathrm{IC},j}=-RT\cdot\ln K_{\mathrm{IC},j} . \tag{S23}$$

Dividing by T and separation of $\Delta S°_{\mathrm{IC},j}$ leads to:

$$S^{\circ}_{\mathrm{IC},j} = \frac{\Delta H^{\circ}_{\mathrm{IC},j}}{T} + R \cdot \ln K_{\mathrm{IC},j} \quad . \tag{S24a}$$

When temperature is substituted with $x = T^{-1}$, following equivalent relation is obtained:

$$S^{\circ}_{\mathrm{IC},x} = x \cdot \Delta H^{\circ}_{\mathrm{IC},x} + R \cdot \ln K_{\mathrm{IC},x} \quad . \tag{S24b}$$

The temperature-dependent molar interconversion entropy (eq. 6) is then set up by simply inserting equations 3 and 5 into equation S24b, so that:

$$\begin{aligned}\Delta S^{\circ}_{\mathrm{IC},x} &= x \cdot \Delta H^{\circ}_{\mathrm{IC},x} + R \ln K_{\mathrm{IC},x} \\ &= -R \cdot x \left(\kappa_1 \kappa_2 e^{\kappa_2 x} - \frac{\kappa_3 \kappa_4}{\kappa_6} \cdot \frac{e^{(x-\kappa_5)/\kappa_6}}{(1+e^{(x-\kappa_5)/\kappa_6})^2} - \frac{\kappa_3(1-\kappa_4)}{\kappa_8} \cdot \frac{e^{(x-\kappa_7)/\kappa_8}}{(1+e^{(x-\kappa_7)/\kappa_8})^2} \right) \\ &\quad + R \left(\ln K_{\mathrm{IC},0} + \kappa_1 \cdot e^{\kappa_2 x} + \frac{\kappa_3 \kappa_4}{1+e^{(x-\kappa_5)/\kappa_6}} + \frac{\kappa_3(1-\kappa_4)}{1+e^{(x-\kappa_7)/\kappa_8}} \right) \quad .\end{aligned} \tag{S25}$$

An expression for the molar interconversion heat capacity $\Delta C^{\circ}_{\mathrm{P,IC},j}$ was found by applying the basic principle given in eq. 11 in Reichenwallner et al.[S14], again by substitution of $T^{-1} = x$:

$$\Delta C^{\circ}_{\mathrm{P,IC},j} = \left(\frac{\partial \Delta H^{\circ}_{\mathrm{IC},j}}{\partial T} \right)_{\mathrm{P}} = -\frac{1}{T^2} \cdot \left(\frac{\partial \Delta H^{\circ}_{\mathrm{IC},j}}{\partial \left(\frac{1}{T}\right)} \right)_{\mathrm{P}} \tag{S26a}$$

and

$$\Delta C^{\circ}_{\mathrm{P,IC},x} = x^2 \cdot \left(\frac{\partial \Delta H^{\circ}_{\mathrm{IC},x}}{\partial x} \right)_{\mathrm{P}} = -Rx^2 \left(\frac{\partial^2 \ln K_{\mathrm{IC},x}}{\partial x^2} \right)_{\mathrm{P}} \quad . \tag{S26b}$$

Therefore, $\Delta H_{\mathrm{IC},x}$ has to be differentiated with respect to x:

$$\frac{\mathrm{d}}{\mathrm{d}x}\left[\Delta H^{\circ}_{\mathrm{IC},x}\right] = -R \left(\underbrace{\frac{\mathrm{d}}{\mathrm{d}x}\left[\kappa_1 \kappa_2 \cdot e^{\kappa_2 x}\right]}_{\text{scheme 1}} + \underbrace{\frac{\mathrm{d}}{\mathrm{d}x}\left[-\frac{\kappa_3 \kappa_4}{\kappa_6} \cdot \frac{e^{(x-\kappa_5)/\kappa_6}}{(1+e^{(x-\kappa_5)/\kappa_6})^2} \right]}_{\text{scheme 3}} + \underbrace{\frac{\mathrm{d}}{\mathrm{d}x}\left[-\frac{\kappa_3(1-\kappa_4)}{\kappa_8} \cdot \frac{e^{(x-\kappa_7)/\kappa_8}}{(1+e^{(x-\kappa_7)/\kappa_8})^2} \right]}_{\text{scheme 3}} \right) . \tag{S27}$$

According to scheme 1 in equation S27 the first bracketed term is given as:

$$\frac{\mathrm{d}}{\mathrm{d}x}\left[\kappa_1 \kappa_2 \cdot e^{\kappa_2 x}\right] = \kappa_1 \kappa_2 \frac{\mathrm{d}}{\mathrm{d}x}\left[e^{\kappa_2 x}\right] = \kappa_1 \kappa_2^2 e^{\kappa_2 x} \, . \tag{S28}$$

The second and third terms are calculated by scheme 3 that utilizes a more interlaced routine:

$$\frac{\mathrm{d}}{\mathrm{d}x}\left[-\frac{\kappa_3\kappa_4}{\kappa_6}\cdot\frac{e^{(x-\kappa_5)/\kappa_6}}{(1+e^{(x-\kappa_5)/\kappa_6})^2}\right]=-\frac{\kappa_3\kappa_4}{\kappa_6}\frac{\mathrm{d}}{\mathrm{d}x}\left[\frac{e^{(x-\kappa_5)/\kappa_6}}{(1+e^{(x-\kappa_5)/\kappa_6})^2}\right]=-\frac{\kappa_3\kappa_4}{\kappa_6}\frac{\mathrm{d}}{\mathrm{d}x}\left[\frac{u(x)}{v(x)}\right], \quad \text{(S29)}$$

where $u_1(x)$, $u_1'(x)$, $u_2(x)$ and $u_2'(x)$ are here identical to the expressions given in scheme 2 (see also eq S14). Additionally, it is:

$$v_1(x)=\left(1+e^{(x-\kappa_5)/\kappa_6}\right)^2=v_2(x)^2 \quad \text{(S30)}$$

and therefore, it follows that:

$$v_2'(x)=u_1'(x)=\frac{\mathrm{d}}{\mathrm{d}x}\left[1\right]+\frac{\mathrm{d}}{\mathrm{d}x}\left[e^{(x-\kappa_5)/\kappa_6}\right]=\frac{e^{(x-\kappa_5)/\kappa_6}}{\kappa_6} \quad \text{(S20b)}$$

with: $$v_1'(x)=\frac{\mathrm{d}}{\mathrm{d}x}\left[v_2(x)^n\right]=n\cdot v_2(x)^{n-1}\cdot v_2'(x)=\frac{2}{\kappa_6}\cdot(1+e^{(x-\kappa_5)/\kappa_6})\cdot e^{(x-\kappa_5)/\kappa_6}. \quad \text{(S31)}$$

The quotient rule gives the expression:

$$\begin{aligned}\frac{\mathrm{d}}{\mathrm{d}x}\left[\frac{u(x)}{v(x)}\right]&=\frac{u_1'(x)\cdot v_1(x)-u_1(x)\cdot v_1'(x)}{v_1(x)^2}\\&=\frac{\frac{1}{\kappa_6}\cdot e^{(x-\kappa_5)/\kappa_6}\cdot\left(1+e^{(x-\kappa_5)/\kappa_6}\right)^2-\frac{2}{\kappa_6}\cdot e^{(x-\kappa_5)/\kappa_6}\cdot\left(1+e^{(x-\kappa_5)/\kappa_6}\right)\cdot e^{(x-\kappa_5)/\kappa_6}}{\left(1+e^{(x-\kappa_5)/\kappa_6}\right)^4}\\&=\frac{e^{(x-\kappa_5)/\kappa_6}}{\kappa_6\cdot\left(1+e^{(x-\kappa_5)/\kappa_6}\right)^2}-\frac{2\cdot e^{2(x-\kappa_5)/\kappa_6}}{\kappa_6\cdot\left(1+e^{(x-\kappa_5)/\kappa_6}\right)^3}\end{aligned} \quad \text{(S32)}$$

and consistently, upon combination of eqs. S29 and S32 it follows that:

$$\begin{aligned}\frac{\mathrm{d}}{\mathrm{d}x}\left[-\frac{\kappa_3\kappa_4}{\kappa_6}\cdot\frac{e^{(x-\kappa_5)/\kappa_6}}{(1+e^{(x-\kappa_5)/\kappa_6})^2}\right]&=-\frac{\kappa_3\kappa_4}{\kappa_6}\frac{\mathrm{d}}{\mathrm{d}x}\left[\frac{u(x)}{v(x)}\right]\\&=-\frac{\kappa_3\kappa_4}{\kappa_6^2}\cdot\frac{e^{(x-\kappa_5)/\kappa_6}}{\left(1+e^{(x-\kappa_5)/\kappa_6}\right)^2}+\frac{2\kappa_3\kappa_4}{\kappa_6^2}\cdot\frac{e^{2(x-\kappa_5)/\kappa_6}}{\left(1+e^{(x-\kappa_5)/\kappa_6}\right)^3}\end{aligned}. \quad \text{(S33a)}$$

The third term in equation S27 can be treated in analogy, so that:

$$\frac{\mathrm{d}}{\mathrm{d}x}\left[-\frac{\kappa_3(1-\kappa_4)}{\kappa_8}\cdot\frac{e^{(x-\kappa_7)/\kappa_8}}{(1+e^{(x-\kappa_7)/\kappa_8})^2}\right]=-\frac{\kappa_3(1-\kappa_4)}{\kappa_8^2}\cdot\frac{e^{(x-\kappa_7)/\kappa_8}}{\left(1+e^{(x-\kappa_7)/\kappa_8}\right)^2}+\frac{2\kappa_3(1-\kappa_4)}{\kappa_8^2}\cdot\frac{e^{2(x-\kappa_7)/\kappa_8}}{\left(1+e^{(x-\kappa_7)/\kappa_8}\right)^3}. \quad \text{(S33b)}$$

Finally, an expression for $\Delta C^{\circ}_{\mathrm{P,IC},x}$ can be put together by using eq S26b:

$$\Delta C^{\circ}_{\mathrm{P,IC},x}=-Rx^2\cdot\left(\kappa_1\kappa_2^2e^{\kappa_2x}-\frac{\kappa_3\kappa_4}{\kappa_6^2}\cdot\frac{e^{(x-\kappa_5)/\kappa_6}}{\left(1+e^{(x-\kappa_5)/\kappa_6}\right)^2}+\frac{2\kappa_3\kappa_4}{\kappa_6^2}\cdot\frac{e^{2(x-\kappa_5)/\kappa_6}}{\left(1+e^{(x-\kappa_5)/\kappa_6}\right)^3}\right.$$
$$\left.-\frac{\kappa_3(1-\kappa_4)}{\kappa_8^2}\cdot\frac{e^{(x-\kappa_7)/\kappa_8}}{\left(1+e^{(x-\kappa_7)/\kappa_8}\right)^2}+\frac{2\kappa_3(1-\kappa_4)}{\kappa_8^2}\cdot\frac{e^{2(x-\kappa_7)/\kappa_8}}{\left(1+e^{(x-\kappa_7)/\kappa_8}\right)^3}\right). \quad \text{(S34)}$$

In order to find characteristic temperatures following equations were used for the plot in **Fig. S6**.

$$T_{\mathrm{P}}=\max\left\{\left|\,\Delta S^{\circ}_{\mathrm{IC},j,6}\cdot\Delta G^{\circ}_{\mathrm{IC},j,6}{}^{-1}\right|\right\} \quad \text{(S35)}$$

$$T_{\mathrm{AD}}=\max\left\{\left|\,\Delta H^{\circ}_{\mathrm{IC},j,6}\cdot\Delta C^{\circ}_{\mathrm{P,IC},j,6}{}^{-1}\right|\right\} \quad \text{(S36)}$$

$$T_{\mathrm{H},i}=\max\left\{\left|\,\Delta S^{\circ}_{\mathrm{IC},j,6}\cdot\Delta H^{\circ}_{\mathrm{IC},j,6}{}^{-1}\right|\right\} \quad \text{(S37)}$$

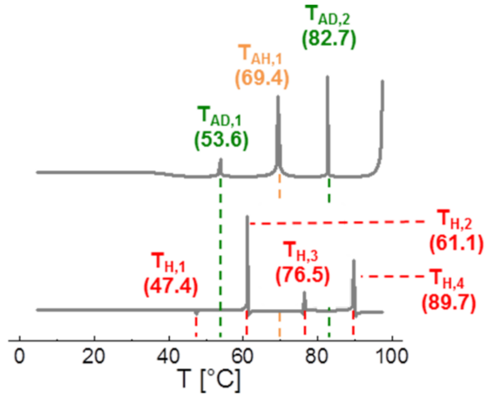

Figure S6 | Characteristic Temperatures from CW EPR data of 16-DSA bound to HSA.
Equations S35 - S37 were used to determine enthalpy compensation temperatures $T_{\mathrm{H},i}$ (red), apolar dehydration temperatures $T_{\mathrm{AD},i}$ (green) and apolar hydration temperature $T_{\mathrm{AH},1}$ (orange).

S7 | Fit Curves from Temperature Stability Shifts in DSC and ln$K_{\mathrm{IC},j}$

For successful predictions of denaturation temperatures $T_{\mathrm{D},i}$ for any 16-DSA loading on HSA at 0.18 mM equivalents, the peak positions of the biphasic thermograms in **Figure 8A** were additionally plotted in **Figure 8B** and fitted with linear ($T_{\mathrm{D},1}$) and exponential functions ($T_{\mathrm{D},2}$). The according parameters were obtained from expressions for $T_{\mathrm{D},1}$:

$$T_{\mathrm{D},1} = T_{\mathrm{D},1,\mathrm{min}} + k_{T_{\mathrm{D},1}} \cdot N_{\mathrm{L}} \qquad \text{S38}$$

and for $T_{\mathrm{D},2}$:

$$T_{\mathrm{D},2} = A_T \cdot e^{-N_{\mathrm{L}}/B_T} + T_{\mathrm{D},2,\mathrm{max}} \quad . \qquad \text{S39}$$

The corresponding results are summarized in **Table S6**.

Table S6 | Fit parameters for denaturation temperature prediction depending on 16-DSA loading

Parameter	Value	SI unit	Physical implication
N_{L}	(variable)	n.a.	no. of 16-DSA per HSA
$k_{\mathrm{TD},1}$	1.2763 ± 0.0583	°C/FA	slope of $T_{\mathrm{D},1}$ increase with N_{L}
$T_{\mathrm{D},1,\mathrm{min}}$	63.58913 ± 0.19760	°C	$T_{\mathrm{D},1}$ for HSA without 16-DSA
A_T	–6.4112 ± 0.1053	°C	–
B_T	0.87377 ± 0.03867	n.a.	–
$T_{\mathrm{D},2,\mathrm{max}}$	76.12554 ± 0.04564	°C	$T_{\mathrm{D},2}$ for HSA saturated with 16-DSA

The ln$K_{\mathrm{IC},j}$ curves that depend on temperature, as well as on the 16-DSA loading ratio are presented in **Figure 8C** and were constructed from fit parameters that can be obtained from Scatchard plots in **Figure 4A** and **S5AB** . Best fits were achieved with exponential fit curves according to following equation:

$$\ln K_{\mathrm{IC},j} = \ln K_{\mathrm{IC},j,0} + A_{\mathrm{K},j} \cdot e^{-N_{\mathrm{L}}/B_{\mathrm{K},j}} \qquad \text{S40}$$

All parameters from these exponential fit curves are shown in **Table S7**.

Table S7 | Fit parameters for ln$K_{\mathrm{IC},j}$ depending on 16-DSA loading and temperature

Parameter	Value	SI unit	Physical implication
N_{L}	(variable)	n.a.	no. of 16-DSA per HSA
ln$K_{\mathrm{IC},25,0}$	–1.34514 ± 0.05279	n.a.	Asymptotic value for $N_{\mathrm{L}} \rightarrow \infty$
ln$K_{\mathrm{IC},37,0}$	–1.10525 ± 0.02677	n.a.	Asymptotic value for $N_{\mathrm{L}} \rightarrow \infty$
$A_{\mathrm{K},25}$	1.63636 ± 0.26177	n.a.	–
$A_{\mathrm{K},37}$	1.50332 ± 0.10046	n.a.	–
$B_{\mathrm{K},25}$	2.20156 ± 0.46068	n.a.	Decay constant of ln$K_{\mathrm{IC},25}$ with N_{L}
$B_{\mathrm{K},37}$	2.28617 ± 0.23768	n.a.	Decay constant of ln$K_{\mathrm{IC},37}$ with N_{L}

S8 | Spin Counting and experimental DEER data for deriving Parameters ln$P_{AB}(r)$ and Δ as Functions of 16-DSA Loading

The basis for extracting 16-DSA loading ratios of HSA is double integration of CW EPR spectra shown in **Figure S7A**. The same samples were subjected to DEER experiments and resulting raw data are shown in **Figure S7B**. This is the foundation of constructing all the plots shown in **Figure 9**.

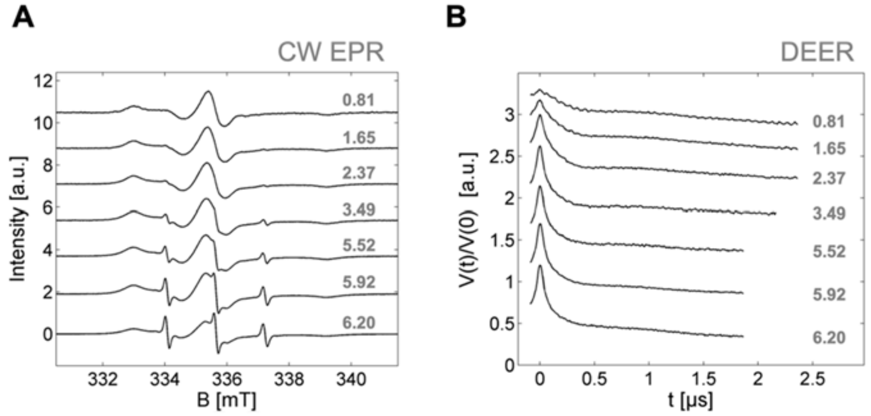

Figure S7 | Spin Counting in CW EPR and raw DEER data of 16-DSA-probed HSA solutiions. A quantitative determination of the co-dissolved 16-DSA molecules in HSA solutions is facilitated by indirect spin-counting utilizing double integration of CW EPR spectra that can be afterwards transferred to ligand concentration-dependent DEER data. (**A**) CW EPR data of HSA loaded in the ratio 0.81 to 6.20 at c_{HSA} = 0.18 mM equivalents. (**B**) Raw time domain DEER data $V(t)/V(0)$ of samples shown in (A).

The ln$P_{AB}(r)$ parameter was introduced to describe the change of characteristic peaks $P_A(r)$ and $P_B(r)$ in loading and temperature-dependent distance distributions from 16-DSA-probed HSA solutions ($P_{AB}(r) = P_A(r)/P_B(r)$). The ln$P_{AB}(r)$ curve in **Figure 9C** can be described with an exponential function of the type:

$$\ln P_{AB}(r) = A_P \cdot e^{-N_{P(r)}/B_P} + \ln P_{AB,0}(r) \qquad \text{S41}$$

Table S8 | Fit parameters from ln$P_{AB}(r)$ in DEER distributions $P(r)$

Parameter	Value	SI unit	Physical implication
$N_{P(r)}$	(variable)	n.a.	no. of 16-DSA per HSA from $P(r)$
A_P	6.83177 ± 0.50259	n.a.	–
B_P	0.99962 ± 0.08071	n.a.	Decay constant for ln$P_{AB}(r)$ with N_L
ln$P_{AB,0}(r)$	–0.45459 ± 0.05489	n.a.	Asymptotic value for ln$P_{AB}(r)$

A shortcut to a more convenient expression can be found when parameter A_P is related to the maximum number of binding sites $N_{T,25}$ in HSA with $A_P/N_{T,25}$ = 0.841 ≈ 17/20, ln$P_{AB,0}(r)$ is replaced by the value –9/20 and parameter B_P is set to 1. After variable separation of $N_{P(r)}$ following expression can be obtained:

$$N_{P(r)} = -B_P \cdot \ln\left(\frac{\ln P_{AB}(r) - \ln P_{AB,0}(r)}{A_P}\right) \qquad \text{(S42)}$$

With these aforementioned assumptions, equation S42 can be simplified to:

$$N_{P(r)} \approx -\ln\left(\frac{\ln P_{AB}(r) + \frac{9}{20}}{\frac{17}{20} \cdot N_{T,25}}\right) = -\ln\left(\frac{20 \cdot \ln P_{AB}(r) + 9}{17 \cdot N_{T,25}}\right) \quad , \qquad \text{(S43)}$$

corresponding to equation 9 of the main text. The Langmuir isotherm analogue expression that was found to reproduce the curve shape of Δ versus N_{Δ} best was fitted with the expression[S15]:

$$\Delta = \frac{\Delta_{max} \cdot b \cdot N_{\Delta}^{(1-c)}}{1 + b \cdot N_{\Delta}^{(1-c)}} \qquad \text{(S44)}$$

where $(1 - c) = \eta$. A similar strategy was used by Sirotkin *et al.*[S16] for modeling enthalpy changes during HSA immersion in aliphatic alcohols. Replacing $(1 - c)$ with η and variable separation for N_{Δ} leads to equation 10 in the main text. The fit parameters of equation S44 can be found in **Table S9**.

Table S9 | Fit parameters from Δ as a function of 16-DSA loading in DEER experiments

Parameter	Value	SI unit	Physical implication
N_{Δ}	(variable)	n.a.	no. of 16-DSA per HSA
Δ_{max}	0.71244 ± 0.04262	n.a.	Maximum attainable Δ-value
b	0.44382 ± 0.11462	n.a.	Langmuir parameter analogue
c	–1.20267 ± 0.52500	n.a.	Exponential stretch parameter

S9 | Supporting References

(S1) Dulbecco, R.; Vogt, M. Plaque Formation and Isolation of Pure Lines with Poliomyelitis Viruses. *J Exp Med* **1954**, *99* (2), 167-182. DOI: DOI 10.1084/jem.99.2.167.

(S2) Wasserman, A. M.; Kasaikin, V. A.; Timofeev, V. P. EPR spin probe and spin label studies of some low molecular and polymer micelles. *Spectrochim Acta A* **1998**, *54* (14), 2295-2308. DOI: Doi 10.1016/S1386-1425(98)00212-1.

(S3) Chin, M.; Somasundaran, P. Enzyme activity and structural dynamics linked to micelle formation: a fluorescence anisotropy and ESR study. *Photochem Photobiol* **2014**, *90* (2), 455-462. DOI: 10.1111/php.12207 From NLM Medline.

(S4) Lewinska, A.; Wilk, K. A.; Jezierski, A. Characterization of the Microenvironments of Alkylamidoamine-N-oxide Surfactant Aggregates by the EPR Spin Labeling Method. *J Solution Chem* **2012**, *41* (7), 1210-1223. DOI: 10.1007/s10953-012-9859-7.

(S5) Rehfeld, S. J.; Eatough, D. J.; Plachy, W. Z. Binding Isotherms for Interaction of 5-Doxyl Stearic-Acid with Bovine and Human-Albumin. *J Lipid Res* **1978**, *19* (7), 841-849.

(S6) Molin, Y. N.; Salikhov, K. M.; Zamaraev, K. I. *Spin Exchange - Principles and Applications in Chemistry and Biology*; Springer Berlin, Heidelberg, 1980.

(S7) Dörfler, H. D. *Grenzflächen und kolloid-disperse Systeme: Physik und Chemie*; Springer-Verlag: Berlin, 2002.

(S8) Fuguet, E.; Ràfols, C.; Rosés, M.; Bosch, E. Critical micelle concentration of surfactants in aqueous buffered and unbuffered systems. *Anal Chim Acta* **2005**, *548* (1-2), 95-100. DOI: 10.1016/j.aca.2005.05.069.

(S9) Reichenwallner, J.; Thomas, A.; Nuhn, L.; Johann, T.; Meister, A.; Frey, H.; Hinderberger, D. Tunable dynamic hydrophobic attachment of guest molecules in amphiphilic core-shell polymers. *Polym Chem-Uk* **2016**, *7* (37), 5783-5798. DOI: 10.1039/c6py01335j.

(S10) Scatchard, G. The Attractions of Proteins for Small Molecules and Ions. *Ann Ny Acad Sci* **1949**, *51* (4), 660-672. DOI: DOI 10.1111/j.1749-6632.1949.tb27297.x.

(S11) Rosenthal, H. E. A Graphic Method for Determination and Presentation of Binding Parameters in a Complex System. *Anal Biochem* **1967**, *20* (3), 525-532. DOI: Doi 10.1016/0003-2697(67)90297-7.

(S12) Reichenwallner, J.; Thomas, A.; Steinbach, T.; Eisermann, J.; Schmelzer, C. E. H.; Wurm, F.; Hinderberger, D. Ligand-Binding Cooperativity Effects in Polymer-Protein Conjugation. *Biomacromolecules* **2019**, *20* (2), 1118-1131. DOI: 10.1021/acs.biomac.9b00016.

(S13) Henning, C.; Stübner, C.; Arabi, S. H.; Reichenwallner, J.; Hinderberger, D.; Fiedler, R.; Girndt, M.; Di Sanzo, S.; Ori, A.; Glomb, M. A. Glycation Alters the Fatty Acid Binding Capacity of Human Serum Albumin. *J Agr Food Chem* **2022**, *70* (9), 3033-3046. DOI: 10.1021/acs.jafc.1c07218.

(S14) Reichenwallner, J.; Schwieger, C.; Hinderberger, D. Probing the Nanoscopic Thermodynamic Fingerprint of Paramagnetic Ligands Interacting with Amphiphilic Macromolecules. *Polymers (Basel)* **2017**, *9* (8). DOI: 10.3390/polym9080324 From NLM PubMed-not-MEDLINE.

(S15) Kapoor, A.; Ritter, J. A.; Yang, R. T. An Extended Langmuir Model for Adsorption of Gas-Mixtures on Heterogeneous Surfaces. *Langmuir* **1990**, *6* (3), 660-664. DOI: DOI 10.1021/la00093a022.
(S16) Sirotkin, V. A.; Borisover, M. D.; Solomonov, B. N. Effect of chain length on interactions of aliphatic alcohols with suspended human serum albumin. *Biophys Chem* **1997**, *69* (2-3), 239-248. DOI: 10.1016/s0301-4622(97)00097-5 From NLM Medline.
